# Association of FXI activity with thrombo-inflammation, extracellular matrix, lipid metabolism and apoptosis in venous thrombosis

**DOI:** 10.1038/s41598-022-13174-5

**Published:** 2022-06-13

**Authors:** Alejandro Pallares Robles, Vincent ten Cate, Andreas Schulz, Jürgen H. Prochaska, Steffen Rapp, Thomas Koeck, Marina Panova-Noeva, Stefan Heitmeier, Stephan Schwers, Kirsten Leineweber, Hans-Jürgen Seyfarth, Christian F. Opitz, Henri Spronk, Christine Espinola-Klein, Karl J. Lackner, Thomas Münzel, Miguel A. Andrade-Navarro, Stavros V. Konstantinides, Hugo ten Cate, Philipp S. Wild

**Affiliations:** 1 Clinical Epidemiology and Systems Medicine, Center for Thrombosis and Hemostasis (CTH), University Medical Center of the Johannes Gutenberg University Mainz, 55131 Mainz, Germany; 2 Preventive Cardiology and Preventive Medicine, Center for Cardiology, University Medical Center of the Johannes Gutenberg University Mainz, 55131 Mainz, Germany; 3 German Center for Cardiovascular Research (DZHK), University Medical Center of the Johannes Gutenberg University Mainz, Partner Site Rhine Main, Mainz, 55131 Germany; 4 Bayer AG, 42113 Wuppertal, Germany; 5 Department of Pneumology, University of Leipzig, 4289 Leipzig, Germany; 6 Department of Cardiology, DRK-Kliniken Westend, 14050 Berlin, Germany; 7 Cardiovascular Research Institute Maastricht (CARIM), Maastricht University Medical Center, Maastricht, 6229 HB The Netherlands; 8 Center for Cardiology – Cardiology I, University Medical Center, Johannes Gutenberg University Mainz, Mainz, 55131 Germany; 9 Institute of Clinical Chemistry and Laboratory Medicine, University Medical Center of the Johannes Gutenberg University Mainz, 55131 Mainz, Germany; 10 Institute of Organismic and Molecular Evolution, Johannes Gutenberg University Mainz, 55128 Mainz, Germany; 11 Center for Thrombosis and Hemostasis (CTH), University Medical Center of the Johannes Gutenberg University Mainz, 55131 Mainz, Germany; 12 Department of Cardiology, Democritus University of Thrace, 68100 Alexandroupolis, Greece

**Keywords:** Translational research, Computational biology and bioinformatics, Biomarkers

## Abstract

Animal experiments and early phase human trials suggest that inhibition of factor XIa (FXIa) safely prevents venous thromboembolism (VTE), and specific murine models of sepsis have shown potential efficacy in alleviating cytokine storm. These latter findings support the role of FXI beyond coagulation. Here, we combine targeted proteomics, machine learning and bioinformatics, to discover associations between FXI activity (FXI:C) and the plasma protein profile of patients with VTE. FXI:C was measured with a modified activated partial prothrombin time (APTT) clotting time assay. Proximity extension assay-based protein profiling was performed on plasma collected from subjects from the Genotyping and Molecular Phenotyping of Venous Thromboembolism (GMP-VTE) Project, collected during an acute VTE event (*n* = 549) and 12-months after (*n* = 187). Among 444 proteins investigated, *N* = 21 and *N* = 66 were associated with FXI:C during the acute VTE event and at 12 months follow-up, respectively. Seven proteins were identified as FXI:C-associated at both time points. These FXI-related proteins were enriched in immune pathways related to causes of thrombo-inflammation, extracellular matrix interaction, lipid metabolism, and apoptosis. The results of this study offer important new avenues for future research into the multiple properties of FXI, which are of high clinical interest given the current development of FXI inhibitors.

## Introduction

Venous thromboembolism (VTE), which encompasses deep vein thrombosis (DVT) and pulmonary embolism (PE), is a common cardiovascular condition and among the leading causes of death in the world^1^. Coagulation factors play a crucial role in the development of the disease. Anticoagulant treatment of the disease is directed against multiple coagulation factors (i.e., heparin and Vitamin K antagonists) or specific proteins located in downstream position in the coagulation cascade (i.e., direct oral factor (F) Xa and thrombin inhibitors), the inhibition of which may result in clinically relevant bleeding events. Recently, the intrinsic coagulation pathway has received much attention due to its apparently lesser involvement in physiological hemostasis and because of its procoagulant properties^2^, rendering this upstream branch of the coagulation pathway a promising potential target for the development of novel anticoagulant drugs. Animal experiments and epidemiological studies indicate that inhibiting FXIa appears to prevent thrombosis without inducing clinically relevant bleeding^3–9^. Novel therapeutic drugs blocking FXI/FXIa have been developed in recent years and new variants are set to appear in the near future. Among them, antisense oligonucleotides (ASO), antibodies and small molecule inhibitors have been tested in phase 1 and 2 clinical trials (review in^2,10^). In this initial, promising phase, more information is needed on how the inhibition of the contact activation system (CAS) and its crosstalk with the immune system could affect disease development and progression in the setting of acute VTE.

The CAS, which involves different serine proteases such as FXII, FXI, prekallikrein (PK) and its cofactor high molecular weight kininogen (HK), is triggered mainly by negatively charged surfaces^11^. Polyphosphates, collagen, glycosaminoglycans (GAGs) and nucleic acids are among the many substances that can activate the CAS^12^. Nevertheless, little emphasis has been placed on how these coagulation factors interact with other systems such as the fibrinolytic system or immune system, as well as what their role is in the setting of thrombo-inflammation. In sepsis models it was established that FXI-deficient (FXI-/-) mice not only have a higher survival rate, but that they also have an attenuated cytokine storm during the acute phase of infection^3–5^. These findings support potential direct or indirect roles for FXI beyond fibrin formation.

Recent advances in proteomics have enabled the targeted, high-throughput relative quantification of a large number of plasma proteins with relevance to pathological molecular processes using an immuno-polymerase chain reaction (PCR)-based multiplex assay (Olink Proteomics, Uppsala, Sweden). Using this technology, the present analysis explored the association between 444 circulating plasma proteins and FXI activity in individuals during an acute VTE event and 12 months post-index event, with the main goal of identifying candidate FXI-related pathways beyond coagulation in the clinical setting of VTE.

## Results

### Characteristics of study sample

Baseline characteristics stratified by FXI:C of study participants included in this analysis are displayed in Table 1. The total number of individuals was *N* = 549 (mean age 59.8 ± 16.5 years). Females were slightly overrepresented in the FXI:C group above the reference range (56.4%). No differences were observed between subgroups regarding age, and prevalence of DVT or PE. VTE-provoking risk factors were equally distributed between groups. There was a higher number of obese individuals in the high FXI:C group.

**Table 1:** Baseline sample characteristics, stratified by categories of FXI:C (%).

	FXI:C (%)		
	Low (< 70%) (*n* = 43)	Normal (70–150%) (*n* = 412)	High (> 150%) (*n* = 94)
Female sex, % (*n*)	41.9 (18)	40.5 (167)	56.4 (53)
Age (years)	55.3 ± 16.9	60.7 ± 16.6	57.8 ± 15.5
Body mass index (kg/m^2^)	26.1 (23.4/30.9)	28.2 (24.7/31.8)	29.2 (25.2/33.2)
Deep vein thrombosis (DVT), % (*n*)	80.5 (33)	81.3 (308)	83.3 (75)
Pulmonary embolism (PE), % (*n*)	62.8 (27)	75.7 (312)	64.9 (61)
**VTE risk factors**			
History of DVT, % (*n*)	27.5 (11)	26.8 (104)	30.1 (28)
History of PE, % (*n*)	8.3 (3)	11.1 (44)	11.8 (11)
Recent immobilization, % (*n*)	12.2 (5)	15.8 (63)	9.7 (9)
Recent surgery, *n* (%)	4.8 (2)	6.2 (25)	2.2 (2)
Recent trauma, *n* (%)	7.3 (3)	3.8 (15)	5.4 (5)
Thrombophilia, *n* (%)	8.3 (3)	4.6 (15)	3.5 (3)
**Cardiovascular risk factors**			
Active smoking, % (*n*)	26.8 (11)	16.1 (61)	20.2 (19)
Arterial hypertension, % (*n*)	48.8 (20)	52.2 (204)	48.9 (45)
Diabetes mellitus, % (*n*)	15.0 (6)	12.0 (47)	15.1 (14)
**Comorbidities**			
Atrial fibrillation, % (*n*)	0 (0)	5.1 (20)	3.3 (3)
Congestive heart failure, % (*n*)	10.3 (4)	4.2 (16)	5.5 (5)
Coronary artery disease, % (*n*)	12.8 (5)	7.5 (29)	3.3 (3)
History of stroke, % (*n*)	2.5 (1)	4.6 (18)	5.5 (5)
Chronic kidney disease, % (*n*)	10.0 (4)	5.2 (20)	7.5 (7)
Chronic liver disease, % (*n*)	11.6 (5)	4.9 (19)	2.2 (2)
Chronic pulmonary disease, % (*n*)	16.7 (7)	11.5 (46)	12.9 (12)
**Medication**			
Vitamin K antagonists, % (*n*)	4.7 (2)	5.6% (23)	8.5% (8)
Heparin group, % (*n*)	44.2 (19)	58.7 (242)	57.4 (54)
Platelet aggregation inhibitors, % (*n*)	23.3 (10)	30.1 (124)	17.0 (16)
Direct Thrombin inhibitors, % (*n*)	4.7 (2)	1.2 (5)	0 (0)
Direct FXa inhibitors, % (*n*)	32.6 (14)	27.4 (113)	20.2 (19)
Cardiovascular medication, % (n)	53.5 (23)	63.3 (261)	47.9 (45)
Contraceptives, % (*n*)	7.0 (3)	1.9 (8)	7.4 (7)
Anti-inflammatory, % (*n*)	7.0 (3)	5.1 (21)	4.3 (4)
**Laboratory markers**			
C-reactive protein (mg/l)	9.26 (2.52/33.97)	15.77 (5.1/44.1)	22.28 (7.29/60.3)
Platelets (/nl)	223.0 (162.0/326.7)	224 (185.7/273)	262 (221.2/300)
APTT (s)	36.40 (31.7/44.2)	33.50 (30.5/37.5)	30.60 (27.9/32.8)
Prothrombin activity (%)	75.51 ± 27.00	82.56 ± 21.16	90.44 ± 19.87

Single numbers indicate mean and standard deviation, or median (interquartile range). Cardiovascular medication comprised cardiac therapy (ATC Code C01), antihypertensives (C02), diuretics (C03), vasoprotective agents (C05), beta blocking agents (C07), calcium channel blockers (C08), agents acting on the renin-angiotensin system (C09) and lipid modifying agents (C010). APTT indicates activated partial thromboplastin time.

In terms of comorbidities, individuals included in the low FXI:C group had higher prevalence of coronary artery disease and chronic liver disease. Further, there were no differences in the distribution of antithrombotic drug use, with the exception of DOAC users, who were slightly overrepresented in the FXI:C group below the reference range, specifically users of FXa inhibitors.

Concerning standard laboratory markers, C-reactive protein (CRP) concentration and platelet count were higher for large FXI:C values. As expected, within the low FXI:C group the APTT was longer, and the prothrombin activity was lower.

### Differences in FXI:C between acute event and 12 months post-index event

**Figure 1:**
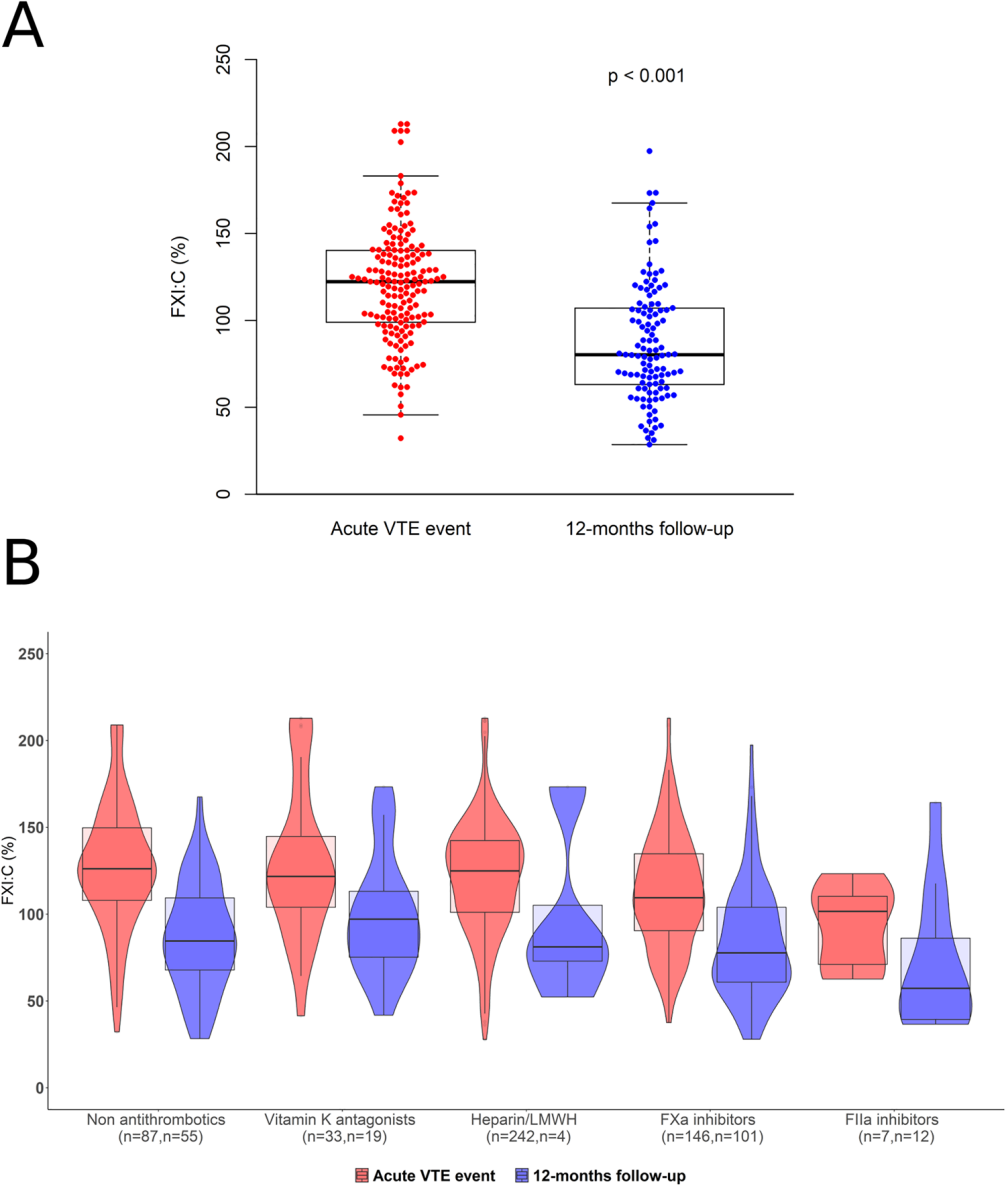
Time-related comparison of factor XI activity (FXI:C) in A, individuals in the acute VTE event and at 12 months of follow-up, and B, stratified by type of anticoagulant. In this figure panel A shows time-differences of FXI:C values available at acute VTE event and 12 months follow-up (*n* = 187). The p-value was derived from a paired t-test. Panel B shows time differences stratified by type of anticoagulant drug, in red for the acute VTE event and in blue for the 12 month follow up time point.

Figure 1A depicts the FXI:C values for those subjects with available measurements in both the baseline and 12 months follow up visits. FXI:C levels were 120.8 ± 33.79% in the acute VTE event and ranged from 32.2 to 212.8%. At 12 months post-index event FXI:C levels were 87.2 ± 34.3% and ranged from 28.5 to 197.3%. FXI:C was significantly higher in the acute event (p < 0.001), reflecting its involvement in the thrombotic event. In Fig. 1B, FXI:C values are displayed stratified by intake of anticoagulants. Individuals under treatment with DOACs had lower FXI:C compared to other anticoagulant users, especially individuals using thrombin inhibitors.

### FXI activity-related plasma proteins

**Figure 2:**
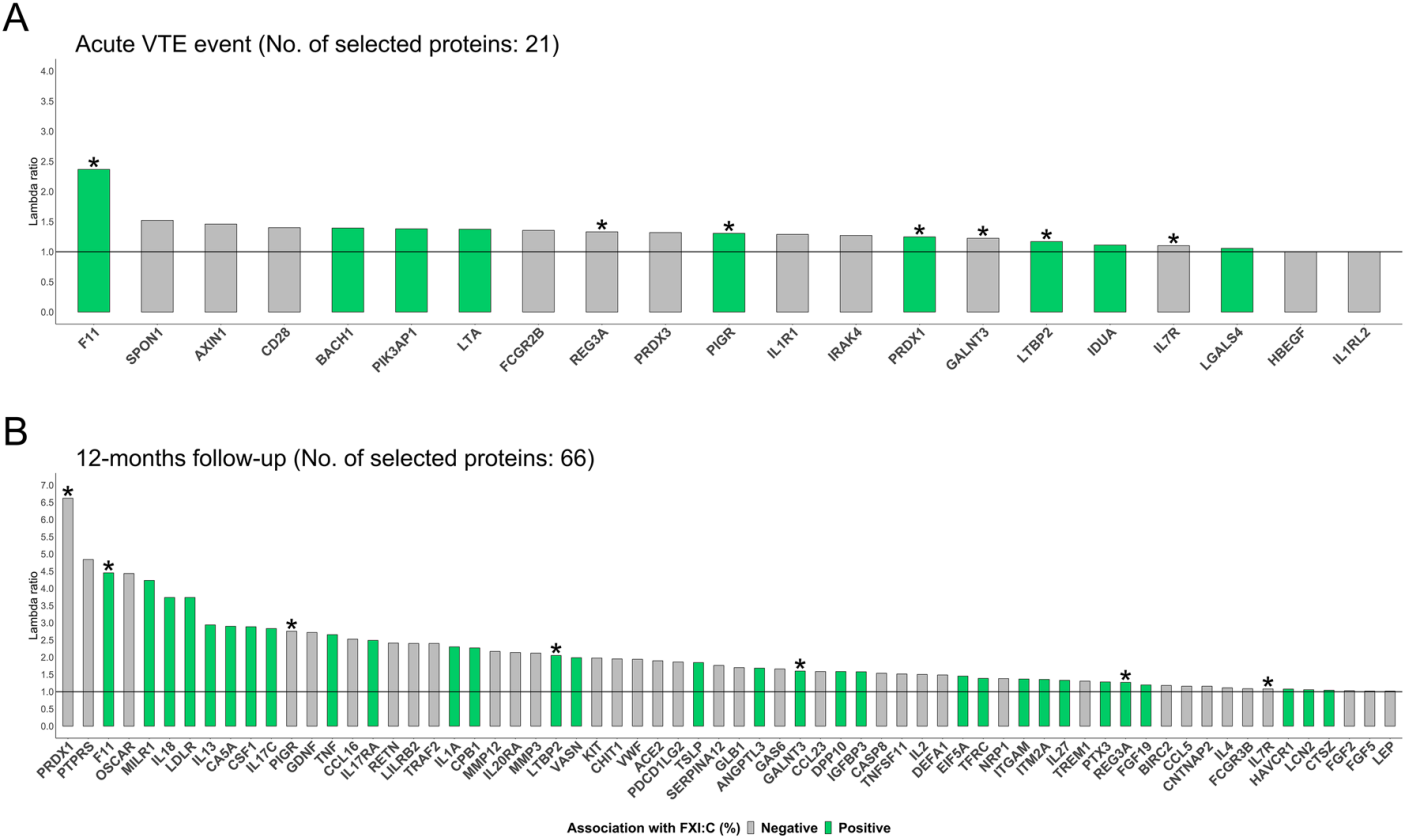
Shared and specific FXI:C-related proteins of the acute VTE event and 12 months after the acute event. This figure displays the shared and specific proteins selected by LASSO-regularized regression models. Panels A and B depict the effect size and association with FXI:C at the acute VTE event and 12 months follow-up, respectively. Proteins selected in both models were shown indicated with a symbol (*).

Among the 444 proteins incorporated in the present analysis, *N* = 21 and *N* = 66 were associated with FXI:C in the LASSO-regularized regression models for the acute VTE event and at 12 months follow-up, respectively. A complete description of covariates and model characteristics can be found in Supplementary Table S2–S3. The seven proteins selected at both time points are denoted with an asterisk in Fig. 2A and B. The robustness of each protein for each model and the association of the protein with the value of FXI:C were also depicted in Fig. 2. Shared proteins include markers of the innate immune system (REG3A, PIGR, IL7R, the transforming growth factor LTBP2, the mitochondrial proteins PRDX1, and the NF-κβ inhibitor GALNT3). The relative concentration of FXI (FXI:ag), was positively correlated with FXI:C at both time points (acute event: Spearman’s ρ = 0.22, 12 months post-index event: ρ = 0.23).

The FXI-related proteins identified only in the acute VTE event were associated with the cell adhesion protein SPON1, cytokine binding (IL1RL2, IL1R1, IL7R, LTA), and the NF-κβ activation IRAK4, and modulation of different immune cells: T-cell activation (CD28), B-cell development (PIK3AP1, FCGR2B), NK-cell activation (SLAMF7), and Th17-cell differentiation (IL17D). In addition, proteins involved in the degradation of extracellular matrix (IDUA), cellular proliferation (AXIN1, BACH1), and mitochondrial proteins (PRDX3) were also identified.

Proteins selected only in the 12-months follow-up model, were characterized by several soluble cytokine receptors (LILRB2, HAVCR1, FCGR3B, MILR1, IL17RA, and IL20RA), and cytokines (IL1A, IL2, IL4, IL18, IL13, IL27, CCL5, CCL16, CCL23, CCL18, and TSLP). Other selected proteins indicated a role for FXI in the modification of the extracellular matrix (MMP3, MMP10, MMP12, PTPRS, GLB1 and CHIT1), cell adhesion (ITGAM, ITMA2, and CNTNAP2), lipid metabolism (LDLR, ANGPTL3, SERPINA12, LEP, IGFBP3, RETN), von Willebrand factor-mediated platelet activation (VWF), growth factors (FGF2, FGF5, FGF19, GDNF, KIT, ENG, and GAS6), neurotrophin family proteins (GDNF, NRP1, and BIRC1), neutrophil degranulation (DEFA1, OSCAR, and LCN2), T-cell activation (PDCD1LG2) including TH17 differentiation (IL17C) and NF-κβ activation (TRAF2, TNF, PTX3 and TNFSF11). Despite the differences regarding the specific proteins selected in both models, the biological processes in which they are involved overlapped between proteomic signatures for both measurement time points. It is noteworthy that associations between FXI:C and the proteins selected were more robust and explained more variance in FXI:C in the follow-up measurement than at baseline, as can be appreciated from the λ ratio and the higher cross-validated R^2^ value, respectively.

### Sensitivity analysis

**Figure 3:**
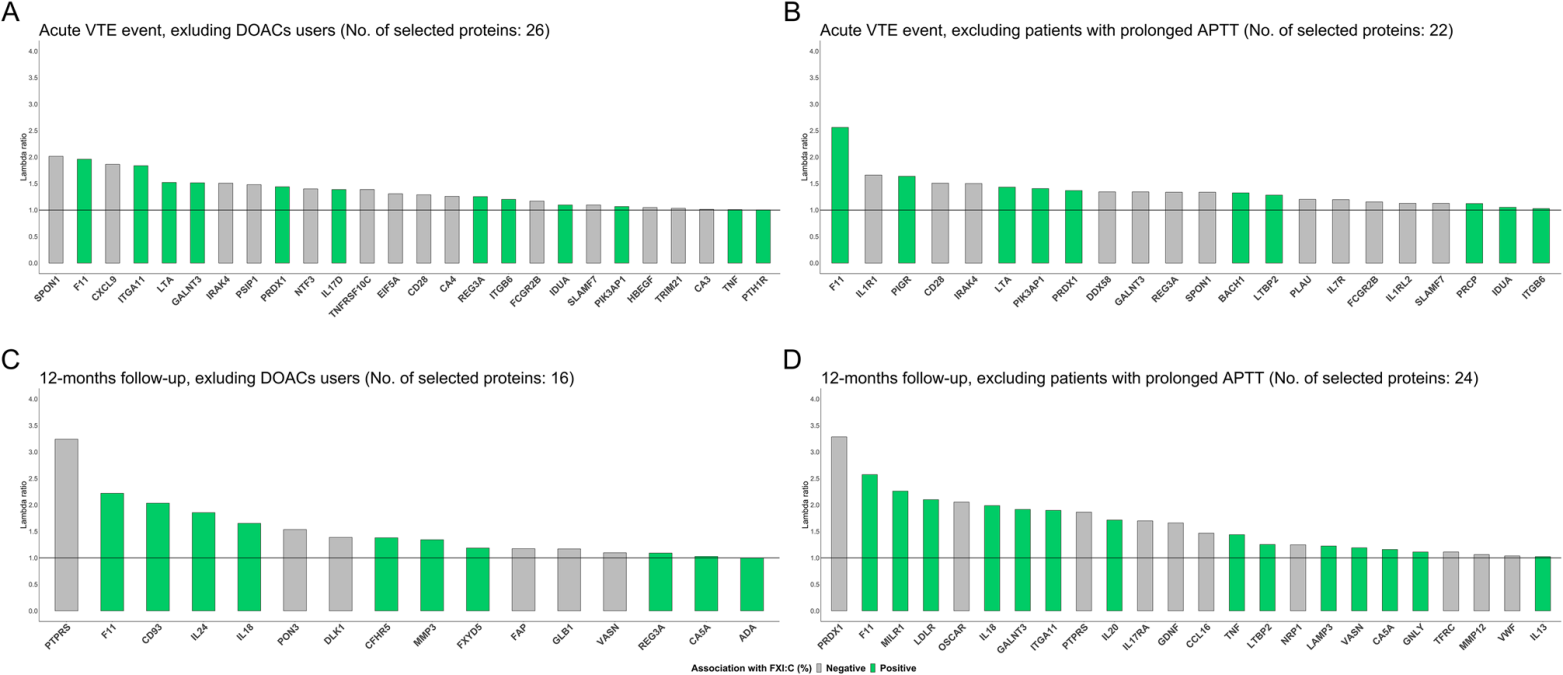
Sensitivity analyses excluding DOAC users, and individuals with normal and high FXI:C (%) and prolonged APTT. This figure shows the proteins selected by LASSO-regularized regression models. Panels A and C depict the size effect and association with FXI:C of the proteins when DOAC users are excluded in the acute VTE event and 12 month follow-up models. Panels B and D depict the selected proteins when individuals with normal and high FXI:C (%) and a prolonged APTT are excluded for each model.

The results of the sensitivity analysis are shown in Fig. 3: when the analysis was restricted to DOAC non-users, similar results were obtained in the acute VTE event model, where 26 proteins were selected, with 12 proteins overlapping between models with and without DOAC users. In the 12 months follow up model the number of proteins selected decreased considerably, where now only 16 proteins were selected and from those only 8 overlapped between models (Supplementary Table S4 and S5). However, 111 observations under DOAC treatment were excluded in this model, and the ability to select proteins using LASSO regression is dependent on sample size. In the second sensitivity analysis, excluding individuals with normal or high FXI:C and an elongated APTT, only 73 and 23 observations were excluded in the acute and 12 months follow up model, respectively (Supplementary Table S6 and S7). In the acute event, the number of proteins selected decreased to 22 with 17 overlapping proteins and in the second model the number of proteins selected decreased as well to 24 proteins with 20 overlapping proteins. Both sensitivity analyses underlined the stability of the proteins selected across models. Finally, a sensitivity analysis was performed to evaluate to what extent chronic liver disease affected the protein signature associated with FXI:C (Supplemental Figure S1). Here, no significant impact could be discerned.

### Pathway enrichment analysis

**Figure 4:**
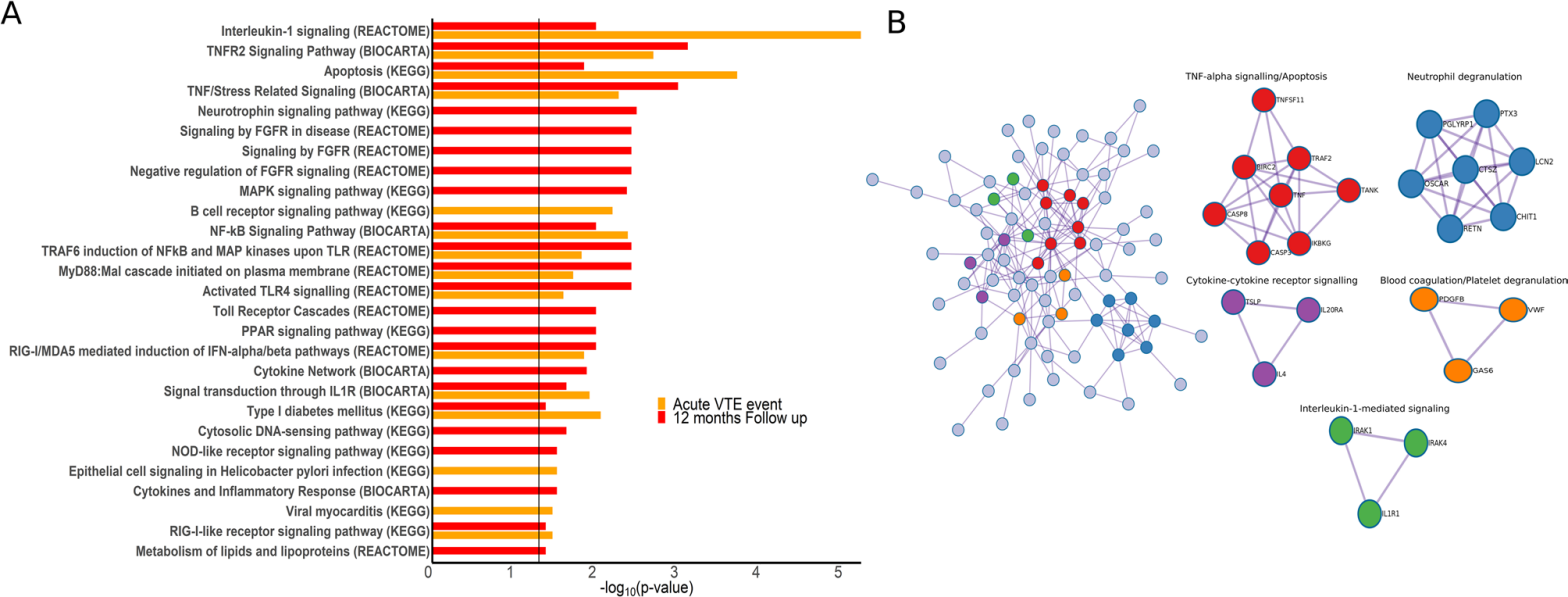
Shared and specific enriched pathways of FXI:C related proteins in the acute VTE event and 12 months follow up (**A**), and protein–protein interaction networks of proteins included in both models (**B**). This figure shows the shared and specific enriched pathways related with FXI:C associated proteins (**A**). The vertical black line depicts the cut-off (*p*-value < 0.05). Orange and red lines depict the *p*-value of a pathway in the acute event and 12 months follow up, respectively. Panel B shows the protein–protein interaction enrichment analysis where only physical interactions are depicted. Networks with 3 or more proteins (densely connected nodes) were color coded and labeled with a functional term based on a Gene Ontology enrichment analysis (https://metascape.org/).

We performed pathway enrichment analysis using established pathway databases in order to compare shared and unique pathways associated with FXI-related proteins in the acute VTE event and 12 months after the index event. The enriched pathways are shown in Fig. 4-A. Several pathways characterized in the acute event were also found in the follow up plasma signature indicating a large overlap in processes. The top enriched pathways found at both time points were Interleukin-1 signalling, apoptosis, TNFR2 signalling, NF-κβ signalling, TNF/Stress related signalling, TRAF6 induction of NF-κβ and MAP kinases upon Toll-like receptor, activated TLR4 signalling and signal transduction through IL1R. Only the B cell receptor signalling pathway and extracellular matrix interaction were enriched specifically in the acute event. Neurotrophin signalling, fibroblast growth factor receptor signalling, signalling by interleukins, Toll receptor cascades, MAPK signalling pathway, RIG-I-like receptor signalling pathway, metabolism of lipids and lipoproteins, cytokine signalling in immune system and NOD-like receptor signalling were found enriched in the follow up phase only. The protein–protein interaction network shown in Fig. 4-B shows distinct networks that are densely connected, limited to curated physical interactions. Five different subnetworks were identified and these were enriched in proteins associated with apoptosis, neutrophil and platelet degranulation, IL1 signaling, and cytokine-cytokine receptor signaling.

## Discussion

Several previous studies have described the association of FXI plasma concentration with cardiovascular risk factors^8^, clinical outcome^7^, routine laboratory biomarkers^13^ and FXI activity^14,15^, but knowledge on the role of FXI beyond coagulation is scarce. To the best of our knowledge, this investigation is the first to relate plasma protein profiles from targeted proteomics to FXI:C in the clinical setting of venous thromboembolism. This study provides new insights regarding the crosstalk between FXI and several plasma proteins that illustrate its diverse roles.

The clinical characteristics associated with FXI:C in this cohort shared similarities with previous results from population-based studies, where individuals with high FXI:C were relatively younger and more often female compared to individuals with normal or low FXI:C^9^. Individuals with lower FXI:C had more comorbidities compared with other groups, particularly increased prevalence of coronary artery disease, chronic liver disease and chronic obstructive pulmonary disease (COPD)^8^. A positive relationship was found between FXI:C and platelet count, which aligns with prior predictions that the amount of thrombin generated via the thrombin-FXIa loop is highly dependent on the platelet count^16^. CRP was increasing with higher FXI:C values and confirmed previous investigation that evaluated the relation between inflammatory markers and FXI levels and the risk of chronic heart disease^13^. FXI:C and FXI:ag measured with PEA technology were found positively correlated, as it has been found in previous studies^15^, being an important predictor in both models.

The methodology used in this study, combining a gold standard coagulation assay, targeted high-throughput proteomics assays and machine learning algorithms, enabled the provision of new insights between the relation of the FXI and immune and cardiovascular proteins. Among the FXI-related proteins that were identified, several were related to the modulation of different cells of the immune system and the immune response to different stimuli. Other selected proteins were involved in processes known to activate FXI through the contact activation system such as extracellular matrix modification^17^, neutrophil degranulation^18^, apoptosis^19^, and the metabolism of different biomolecules^20^.

### Established FXI-related proteins corroborated by this study

Several studies have shown the interaction of FXI with its known targets within the coagulation system (thrombin, FXIIa, HK and FIX) and with other proteins beyond the coagulation system, elucidating the reactivity of this serine protease.

Beyond coagulation, FXI was also found to be associated with proteins belonging to the low-density protein receptor (LDLR) family, such as the apolipoprotein E receptor 2 (APOER2) that acts blocking the binding of FXI or FXIa to the platelet surface^21^. FXI has a disulphide bond that binds together both subunits. Redox changes in the disulphide bonds can influence the activation of the zymogen^22^. Giannakopoulos et al. showed that thiol-disulfide exchange with FXI can be regulated by oxidoreductases enzymes^23^. PRDX1 is a thiol-specific peroxidase that plays a role in cell protection against oxidative stress and is able to reduce disulphide bonds^24^. FXI has two clusters of basic amino acids in the apple domain 3 (A3) and in the catalytic domain that interact with different polyanions like the anticoagulant glycosaminoglycan heparin^25^. Heparin accelerates the inhibition of FXIa by antithrombin and C1-inhibitor. Different plasma proteins present heparin-binding domains allowing their interaction with heparin by modulating its concentration and ability to enhance the inhibition of different coagulation proteases. HB-EGF, LTBP2 and PTPRS possess heparin-binding domains and have a high affinity to bind heparin and heparan sulphate proteoglycans^26,27^. Misfolded proteins tend to form aggregates that can interact with different systems including the CAS, mainly via FXIIa leading to a bradykinin-mediated inflammatory response^28^. It is not clear whether activation of the intrinsic pathway by aggregate amyloid-β proteins can contribute to FXI activation^29^. Interestingly, the generation of kallikrein contributes to FIX activation in conditions of FXI deficiency, therefore variation in FXI may allow bypassing effects of PKa in downstream clotting^30^. SPON1 is a cell adhesion extracellular matrix protein that also exerts an inhibitory effect on the production of amyloid-β proteins due to inhibition of beta-site amyloid precursor protein cleaving enzyme 1 (BACE1), resulting in a downregulation of amyloid-β protein formation and, therefore, potentially resulting in an attenuated activation of the CAS^31^.

Despite these examples of known interactions between FXI and plasma proteins that were confirmed with the results of this investigation, other proteins included in the panel that interact with FXI or FXIa such as RARRES2^32^, PAI-1^33^, GP1BA^34^, were not found to be associated.

### FXI-related pathways

**Figure 5:**
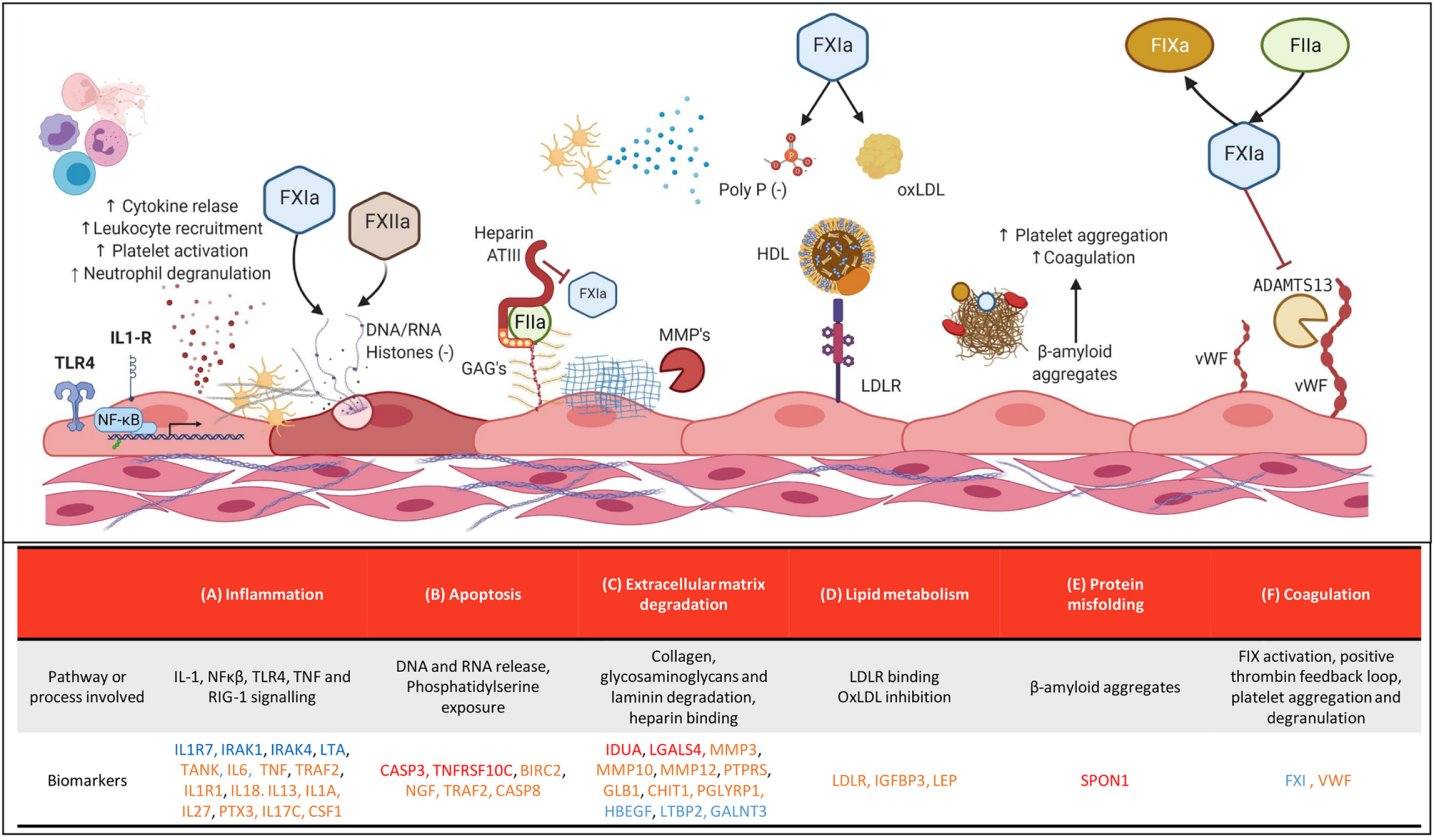
Summary representation of the main pathways and proteins related with FXI:C in the setting of VTE. This summary figure illustrates the main processes and biomarkers related to FXI:C associated proteins. Proteins highlighted in red were associated in the acute VTE event, in orange with the 12-month post-index event and in blue at both time points. (**A**) Cytokine receptors are located on the surface of endothelial cells, enhance the expression of proinflammatory proteins and their release, promote leukocyte recruitment, platelet activation, neutrophil degranulation and netosis (DNA/RNA and histones released), and local inflammation, which could act as a procoagulant environment. (**B**) During apoptosis, apoptotic bodies are released which, together with phosphatidylserine exposure, support a negatively charged surface that can contribute to the activation of FXII and FXI, resulting in increased coagulation activity. (**C**) Extracellular matrix components such as glycosaminoglycans and collagen support a procoagulant surface against different coagulation factors and cell types. Extracellular matrix turnover by proteins involved in degradation of matrix components is a relevant process that prevents the binding and aggregation of platelets and proteins involved in coagulation. (**D**) The products of LDL oxidation (OxLDL) interact with various components of the coagulation system, but are also capable of promoting platelet activation and endothelial dysfunction. (**E**) β-Amyloid protein aggregation may facilitate the aggregation of platelets and coagulation factors, which may lead to thrombus formation in small vessels. (**F**) FXI has the ability to cleave ADAMTS13, which promotes activation of the intrinsic coagulation cascade and platelet aggregation via vWF. Created with BioRender.com.

By identifying shared and distinct proteomic signatures at two time points (i.e. the acute phase of the VTE and 12 months post-index event), a more detailed picture of the role of FXI under various circumstances emerged. In this way, distinct and shared pathways were discovered, which are briefly discussed below (Fig. 5).

### Crosstalk of FXI with the immune system

Of the inflammatory molecules implicated in the crosstalk between inflammation and coagulation, interleukin (IL)-1 plays a known role in the development of thrombosis^35^. IL-1α and IL-1β exert their action by binding to IL-1R1. Upon binding, protein complexes are assembled involving proteins such as MyD88, IRAK1/4 and TRAF6 (e.g. the so called Myddosome between MyD88 and IRAK-4)^36^ which initiate a series of reactions that trigger different transcription factors (via NF-κβ-dependent and independent mechanisms) to induce the expression of TLR/IL-1R-responsive genes that mediate the production of inflammatory mediators^35^. While several single nucleotide polymorphisms (SNPs) found in the IL-1 cytokine family have been associated with a reduced or increased risk of DVT, they have demonstrated only limited diagnostic value^37^. Animal studies additionally support the relationship between elevated FXI:C and a stimulated expression of inflammatory mediators^6^.

Complementary to the IL-1-signalling pathway, NF-κβ is an inducible transcription factor that regulates the expression of genes involved in the inflammatory response, cell cycle progression, inhibition of apoptosis and angiogenesis^38^. Among the inflammatory and pro-thrombotic proteins positively regulated by the NF-κβ pathway are C-reactive protein, IL-6, TNF-α, IL-1β, MMP-9, vascular endothelial growth factor (VEGF), TF and P-selectin^39^. Further, Peroxiredoxin-1 (PRDX1) is a ubiquitously expressed enzyme that reduces peroxide levels. In addition to its known protective activity, PRDX1 was recently recognized as a damage-associated molecular pattern (DAMP) molecule involved in the activation of TLR 2–4 that can also induce the expression of IL-1β, IL-6 and TNF-α through NF-κβ signalling^24,40^.

TLRs are a family of receptors expressed mainly in immune cells that are activated by pathogen- or damage-associated patterns (PAMPs or DAMPs)^41^. Recently, it was demonstrated that TLR4 is expressed on the platelet surface^42^. Platelets activated via TLR4 may also mediate the formation of neutrophil extracellular traps (NETs), due to the release of vWF, platelet factor 4 (PF4), and thromboxane (TXA2), molecules that increase the release of DNA traps^43^. NETs can consequently activate platelets, promoting the aggregation and recruitment that can induce thrombin generation by the activation of TLR4 and that is amplified via the CAS in conditions of systemic inflammation^44^. The importance of this connection further underlines the close relationship between the immune response and the hemostatic system.

Although there is a direct interrelation between FXI(a) and inflammation, it is not understood in detail. In this investigation we have found several markers associated with FXI:C that have a role in T-cell activation and chemotaxis (CD28, CCL16, CCL23, CCL5, TSLP), inflammatory mediators (IL1A, PTX3, IL18, IL17C, IL27), and host defense proteins (REG3A, DEFA1, CHIT1 and CTSZ). Whether pharmacological FXIa inhibition could hamper or alter the different processes of the immune system in different disease settings is a relevant question that deserves further scientific scrutiny in follow-up investigations.

### Other FXI activity-related pro-thrombotic molecular mechanisms

In addition to the above, several other mechanisms with direct links to thrombosis appear to be stimulated by FXI:C, based on the results of this investigation. One such mechanism is extracellular matrix reorganization. The extracellular matrix (ECM) is a complex structure composed of proteoglycans (heparan sulphate, chondroitin sulphate and keratan sulphate), hyaluronic acid, collagens, elastin and cell adhesion proteins such as fibronectin and laminin^45^. Most of these proteins interact with negatively charged components that can promote FXII and FXI activation or inhibition, additionally via HK mediation^46^. The degradation or isolation of extracellular matrix by different matrix degradation proteins after tissue damage and therefore subendothelial exposure can limit the binding of platelets and coagulation proteins and prevent thrombus formation.

Another mechanism of interest identified by this investigation is the link to lipid metabolism. This has a known relevant impact in arterial thrombosis, but may also contribute to venous thrombosis. Oxidation of low-density lipoproteins (OxLDL) results in the release of oxidized cholesterol and phospholipids among other fatty acid products. Different studies have shown that OxLDL can alter the coagulation cascade in two independent ways: via inhibiting plasma proteins of the intrinsic pathway FVIII, FIX and FXI ^47^ and by stimulating the production of TF by endothelial cells^48^. Furthermore, the platelet surface protein CD36 can interact with OxLDL, promoting platelet activation, providing another mechanism through which lipid metabolism can lead to thrombosis^49^.

Finally, this investigation has identified connections between FXI:C and several apoptosis-related proteins. Apoptosis is a common process in various pathological settings, as the clearance of cellular debris is important in order to avoid major complications. Diverse links between apoptosis and coagulation have already been elucidated. During apoptosis, phosphatidylserine (PS) is exposed, which can cleave and activate FXII in presence of HK and PK, promoting tenase (FXa/FVa) formation and blood clotting^19^.

### Limitations

While the proteins analyzed for this study fall within the area of thrombosis and inflammation, the specific set of included proteins was based on prior knowledge and the design of the different panels used in this targeted proteomics platform. This inherently limits the associations that can be detected between FXI:C and the plasma proteome. Future studies using untargeted proteomics approaches such as mass spectrometry could contribute further knowledge about the associations of FXI and its roles beyond coagulation.

The vast majority of the VTE patients received anticoagulants prior to blood sampling on suspicion of acute VTE, which could affect coagulation activity and thereby might have influenced our results. Although most of our markers did not seem to be affected by anticoagulant use, such effects cannot be entirely ruled out. Although the use of LMWH and DOAC neutralizing agents like DOAC stop would theoretically correct an anticoagulant effect on the FXI:C assay, this procedure is not yet widely established in clinical laboratories and therefore was not pursued in this instance.

The cellular source of the proteins identified when investigating blood plasma from the systemic circulation is unknown. While hypotheses about the implicated pathways can be made based on the large number of proteins included in the analysis, further experiments are needed to determine the contributions of individual cell types to the proteomic profile.

## Conclusions

This study reports the associations between FXI:C and an extensive set of plasma proteins in the clinical setting of venous thrombosis by exploratory high-throughput targeted proteomics. The identified FXI-associated proteins are involved in several known pathways related to specific causes of thrombo-inflammation such as IL-1, NF-κβ and TLR4 signalling pathways, in addition to relevant mechanisms including apoptosis and tissue remodelling. Given its complex interactions with the immune system, inhibition of FXI could induce a host of pleiotropic effects beyond inhibition of coagulation. Future studies should validate and determine the clinical relevance of these findings, especially to define the optimal clinical use of FXI inhibitors.

## Methods

### Participants

The data used for this investigation is part of the *Genotyping and Molecular Phenotyping of Venous ThromboEmbolism* (GMP-VTE) Project, an investigator-initiated, multi-center prospective cohort study with comprehensive biobanking situated in Germany. The background, rationale and description of the design have been described in detail elsewhere^50^. The GMP-VTE project includes VTE patients from two previously established prospective cohort studies, VTEval (ClinicalTrials.gov identifier: NCT02156401) and FOCUS Bioseq (German clinical trials registry identifier: DRKS00005939). Both studies were approved by the independent ethics committee of each participating study site (Rhineland-Palatinate Medical Association reference numbers 837.320.12 [8421-F] and 837.440.13 [9125]). The studies were designed and executed in accordance with local legal and regulatory requirements, including the General Data Protection Regulation (EU 2016/679) and the Declaration of Helsinki (2013, 7th revision). Written informed consent was obtained prior to study enrolment including the consent for biomaterial and blood sampling, genetic analysis, and sharing of data with research partners.

### Measurement of circulating plasma proteins

EDTA plasma samples from venous blood obtained from study participants were analyzed using the proximity extension assay (PEA) technology (Olink Proteomics, Uppsala, Sweden). This targeted proteomics technology combines oligonucleotide-labelled antibodies and real time polymerase chain reaction (PCR) amplification in a multiplexed platform, producing normalized protein expression (NPX) values^51^. Five of these Target 96 immuno-PCR panels were utilized (trading names: Cardiometabolic, Cardiovascular II, Cardiovascular III, Inflammation and Immune response panel), resulting in the relative quantification of 444 unique proteins. Measurements were performed in samples collected at two time points, taken during the acute event and at a 12 months follow-up. A detailed description of the protein profiling methodology and quality control procedures applied in the GMP-VTE Project is provided elsewhere^50^.

### Measurement of FXI activity

FXI activity (FXI:C) was measured using both citrate FXI plasma-deficient substrate and patient blood plasma using a Siemens BCS® XP system (Siemens Healthcare GmbH, Erlangen, Germany), a modified activated partial prothrombin time (APTT) clotting time assay that uses recombinant human thromboplastin in a coagulometric method. Pathromtin® SL was used as FXII activating reagent to trigger the reaction. The time to clot formation is measured optically, and the FXI concentration was calculated using a FXI calibration curve.

### Statistical analysis

To prevent confounding by the known proinflammatory protein profile associated with cancer, patients with active malignancy were excluded from this analysis. Active malignancy was defined as any non-remissive or unresolved cancer, including those under active treatment. Further, patients with severe FXI deficiency (FXI:C < 20%) were excluded from the analysis. Baseline characteristics of individuals were described as relative and absolute frequencies for categorical variables and mean and standard deviation for continuous variables. Non-normally distributed variables were described by median and interquartile range (IQR). Baseline characteristics were shown stratified by subgroups of FXI:C, corresponding to normal activity (70–150%), low activity (< 70%) and high activity (> 150%).

Protein expression levels following non-normal distributions were transformed to normality using square root or natural logarithmic transformations. To identify proteins that have linear and non-linear associations with FXI:C, multivariable linear regression models with least absolute shrinkage and selection operator (LASSO) regularization and fractional polynomial transformations of all proteins were used. The computed models were adjusted for covariates that could potentially alter both FXI:C and protein expression levels: sex, age, body mass index (BMI), VTE-provoking transient risk factors (e.g. pregnancy, recent surgery or trauma, recent immobilization, recent long-distance travel), comorbidities and medication. A complete list of all model covariates is provided in Supplemental Table S1-7. The regularization parameter (λ) was selected using tenfold cross-validation (CV), minimizing the mean squared error (MSE) to enhance the generalizability of the model. To rank the selected features, the lambda ratio (LR) was used. The LR is a scale-invariant measure that indicates the relevance of the proteins based on how robustly predictive they are. It is calculated by computing the ratio of the λ at which a protein was included in the model to the optimal λ selected by cross-validation.

Additionally, sensitivity analyses were performed in order to test the stability of the results: (1) a first sensitivity analysis restricted the analysis to non-users of direct oral anticoagulants (DOAC) due to their specific impact on the coagulation system and the APTT assay; (2) a second sensitivity analysis excluded individuals with normal or high FXI:C and an elongated APTT. These two sensitivity analyses were performed in accordance with recommendations given in Salomon et al.^7^.

All data pre-processing and analysis were conducted using the statistical software package R, version 3.5.3 (R Foundation for Statistical Computing, Vienna, Austria).

### Bioinformatic analysis

A gene set pathway enrichment analysis was performed using the XGR R package^52^ and the annotations of different functional databases including the Kyoto Encyclopedia of Genes and Genomes (KEGG)^53^, Reactome^54^, and Biocarta^55^. In this analysis, proteins selected by the two regularized regression models, as well as proteins with high correlation (Spearman’s ρ > 0.80) with the selected proteins were used (Supplementary Table S8and S9). As background for the enrichment analyses, only those genes corresponding with the measured proteins were used.

**Figure 6:**
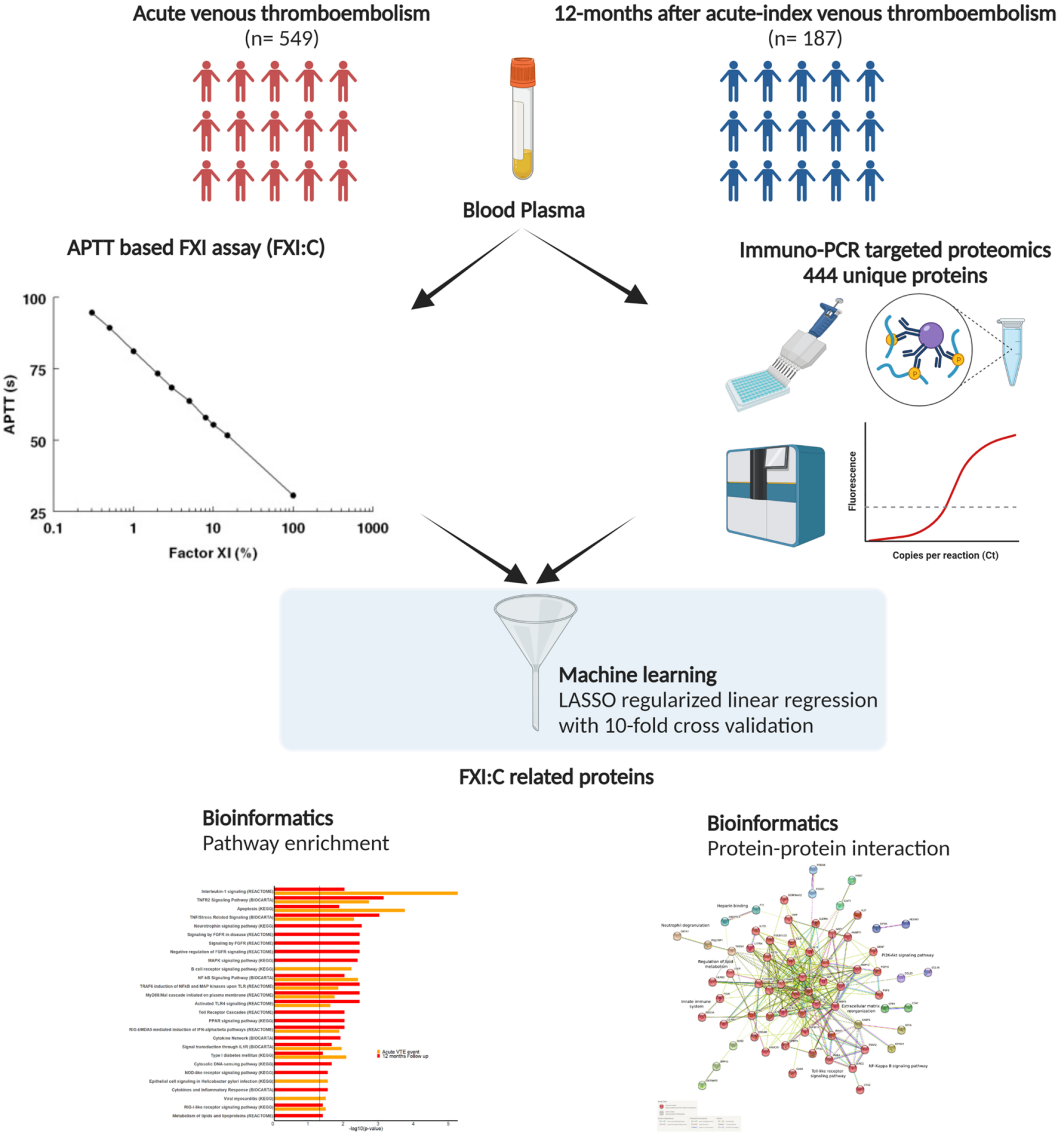
Methods overview and flow chart of the study investigating FXI:C related proteins.

In order to facilitate the interpretation of results, protein–protein interaction networks were generated with the in-browser tools STRING (http://string-db.org) and Metascape (https://metascape.org/). Two different networks were generated, one for the acute VTE event and one for proteins selected for the 12 months-follow up time point. Node colors were based on labels derived by the Markov clustering algorithm^56^ with the default inflation parameter value to enable the representation of functional protein clusters (Supplementary Figure S2-3). To identify densely connected subnetworks forming only physical interactions, the Molecular Complex Detection (MCODE)^57^ algorithm was applied. Each MCODE network was assigned a unique color. Gene ontology (GO) enrichment analysis was applied to each MCODE network to assign “meanings” to the network component. A flowchart and method overview for this investigation is shown in Fig. 6.

## Supplementary Information

Supplementary Information.

## Data Availability

Data are not made available for the scientific community outside the established and controlled workflows and algorithms. To meet the general idea of verification and reproducibility of scientific findings, we offer access to data at the local database in accordance with the ethics vote upon request at any time. Interested researchers make their requests to the coordinating principal investigator of the GMP-VTE project (Dr. Philipp S. Wild; philipp.wild@unimedizin-mainz.de). The R code used for the analyses reported in this paper is available upon request.
