# Supplementary material for: Association of FXI activity with thrombo-inflammation, extracellular matrix, lipid metabolism and apoptosis in venous thrombosis
Source: Sci Rep. 2022 Jun 13;12:9761. doi: 10.1038/s41598-022-13174-5 (PMC9192691; doi:10.1038/s41598-022-13174-5)
Supplement: Supplementary file 1 — Supplementary Information. [file 41598_2022_13174_MOESM1_ESM.docx]

**Association of FXI activity with thrombo-inflammation, extracellular matrix, lipid metabolism and apoptosis in venous thrombosis**

**Running title:** FXI activity and the plasma protein profile in VTE

Alejandro Pallares Robles, MSc^1^; Vincent ten Cate, PhD^1,2^; Andreas Schulz, PhD^2^; Jürgen H. Prochaska, MD^1,2,3^; Steffen Rapp, PhD^2^; Thomas Koeck, PhD^2,3^; Marina Panova-Noeva, MD MSc PhD^1,2^; Stefan Heitmeier, PhD^4^; Stephan Schwers, PhD^4^; Kirsten Leineweber, PhD^4^; Hans-Jürgen Seyfarth, MD^5^, Christian F. Opitz, MD^6^; Henri Spronk, PhD^7^; Christine Espinola-Klein, MD^8^; Karl J. Lackner, PhD^9^; Thomas Münzel, MD^8^; Miguel A. Andrade-Navarro, PhD^10^; Stavros V. Konstantinides, MD PhD^11,12^; Hugo ten Cate, MD PhD^7^; *Philipp S. Wild, MD MSc^1,2,3^

1. Clinical Epidemiology and Systems Medicine, Center for Thrombosis and Hemostasis (CTH), University Medical Center of the Johannes Gutenberg University Mainz, Mainz, 55131 Germany;
2. Preventive Cardiology and Preventive Medicine, Center for Cardiology, University Medical Center of the Johannes Gutenberg University Mainz, Mainz, 55131, Germany;
3. German Center for Cardiovascular Research (DZHK), Partner Site Rhine Main, University Medical Center of the Johannes Gutenberg University Mainz, 55131, Germany;
4. Bayer AG, Wuppertal, 42113, Germany;
5. Department of Pneumology, University of Leipzig, Leipzig, 4289, Germany;
6. Department of Cardiology, DRK-Kliniken Westend, Berlin, 14050, Germany;
7. Cardiovascular Research Institute Maastricht (CARIM), Maastricht University Medical Center, Maastricht, 6229 HB, the Netherlands;
8. Center for Cardiology – Cardiology I, University Medical Center of the Johannes Gutenberg University Mainz, 55131, Germany;
9. Institute of Clinical Chemistry and Laboratory Medicine, University Medical Center of the Johannes Gutenberg University Mainz, Mainz, 55131, Germany;
10. Institute of Organismic and Molecular Evolution, Johannes Gutenberg University Mainz, Mainz, 55128, Germany;
11. Center for Thrombosis and Hemostasis (CTH), University Medical Center of the Johannes Gutenberg University Mainz, Mainz, 55131, Germany;
12. Department of Cardiology, Democritus University of Thrace, Alexandroupolis, 68100, Greece.

Collaborators: R. Ewert (Greifswald), H.A. Ghofrani (Gießen), M. Halank (Dresden), M. Held (Würzburg), F.J. Meyer (München Bogenhausen), G. Weißer (Mainz).

**Correspondence**

Philipp S. Wild, MD, MSc

Professor of Clinical Epidemiology

University Medical Centre Mainz of the Johannes Gutenberg University Mainz,

Langenbeckstr. 1, 55131 Mainz, Germany;

Phone: +49 6131 17 7163; Fax: +49 6131 17 8460;

Email: philipp.wild@unimedizin-mainz.de

**Table of contents**

| Identifier | Title | Page number |
| --- | --- | --- |
| ST1 | Protein names and abbreviations for all measured proteins | 3 |
| ST2 | Model estimates for LASSO-regularized lineal regression. Baseline model. | 13 |
| ST3 | Model estimates for LASSO-regularized lineal regression. 12 months follow up model. | 15 |
| ST4 | Sensitivity analysis excluding DOACs users. Baseline model. | 18 |
| ST5 | Sensitivity analysis excluding DOACs users. 12 months follow up model. | 19 |
| ST6 | Sensitivity analysis excluding individuals with elongated APTT and normal or high FXI:c. Baseline model. | 20 |
| ST7 | Sensitivity analysis excluding individuals with elongated APTT and normal or high FXI:c. 12 months follow up model. | 21 |
| ST8 | Proteins with high correlations (Spearman’s rank correlation coefficient>0.8) with proteins selected in the baseline model. | 22 |
| ST9 | Proteins with high correlations (Spearman’s rank correlation coefficient>0.8) with proteins selected in the 12 months follow up model. | 25 |
| SF1 | Factor XI activity (FXI:C) and expression of the associated protein signature in patients with and without chronic liver disease. | 26 |
| SF2 | String network analysis of FXI-related proteins at baseline | 27 |
| SF3 | String network analysis of FXI-related proteins at 12 moths follow up | 28 |

**Supplemental Table 1.** Protein names and abbreviations for all measured proteins (N=444)

| Abbreviation | Full name |
| --- | --- |
| 4E-BP1 | Eukaryotic translation initiation factor 4E-binding protein 1 (4E-BP1) |
| ACE2 | Angiotensin-converting enzyme 2 (ACE2) |
| ADA | Adenosine Deaminase (ADA) |
| ADAM-TS13 | A disintegrin and metalloproteinase with thrombospondin motifs 13 (ADAM-TS13) |
| ADM | ADM (ADM) |
| AGRP | Agouti-related protein (AGRP) |
| ALCAM | CD166 antigen (ALCAM) |
| AMBP | Protein AMBP (AMBP) |
| ANG | Angiogenin (ANG) |
| ANG-1 | Angiopoietin-1 (ANG-1) |
| ANGPTL3 | Angiopoietin-related protein 3 (ANGPTL3) |
| AOC3 | Membrane primary amine oxidase (AOC3) |
| AP-N | Aminopeptidase N (AP-N) |
| APOM | Apolipoprotein M (APOM) |
| AREG | Amphiregulin AR (AREG) |
| ARNT | Aryl hydrocarbon receptor nuclear translocator (ARNT) |
| ARTN | Artemin (ARTN) |
| AXIN1 | Axin-1 (AXIN1) |
| AXL | Tyrosine-protein kinase receptor UFO (AXL) |
| AZU1 | Azurocidin (AZU1) |
| BACH1 | Transcription regulator protein BACH1 (BACH1) |
| Beta-NGF | Beta-nerve growth factor (Beta-NGF) |
| BIRC2 | Baculoviral IAP repeat-containing protein 2 (BIRC2) |
| BLM hydrolase | Bleomycin hydrolase (BLM hydrolase) |
| BMP-6 | Bone morphogenetic protein 6 (BMP-6) |
| BNP | Natriuretic peptides B (BNP) |
| BTN3A2 | Butyrophilin subfamily 3 member A2 (BTN3A2) |
| C1QTNF1 | Complement C1q tumor necrosis factor-related protein 1 (C1QTNF1) |
| C2 | Complement C2 (C2) |
| CA1 | Carbonic anhydrase 1 (CA1) |
| CA3 | Carbonic anhydrase 3 (CA3) |
| CA4 | Carbonic anhydrase 4 (CA4) |
| CA5A | Carbonic anhydrase 5A, mitochondrial (CA5A) |
| CASP-3 | Caspase-3 (CASP-3) |
| CASP-8 | Caspase-8 (CASP-8) |
| CCL11 | Eotaxin (CCL11) |
| CCL14 | C-C motif chemokine 14 (CCL14) |
| CCL15 | C-C motif chemokine 15 (CCL15) |
| CCL16 | C-C motif chemokine 16 (CCL16) |
| CCL17 | C-C motif chemokine 17 (CCL17) |
| CCL18 | C-C motif chemokine 18 (CCL18) |
| CCL19 | C-C motif chemokine 19 (CCL19) |
| CCL20 | C-C motif chemokine 20 (CCL20) |
| CCL23 | C-C motif chemokine 23 (CCL23) |
| CCL24 | C-C motif chemokine 24 (CCL24) |
| CCL25 | C-C motif chemokine 25 (CCL25) |
| CCL28 | C-C motif chemokine 28 (CCL28) |
| CCL3 | C-C motif chemokine 3 (CCL3) |
| CCL4 | C-C motif chemokine 4 (CCL4) |
| CCL5 | C-C motif chemokine 5 (CCL5) |
| CD163 | Scavenger receptor cysteine-rich type 1 protein M130 (CD163) |
| CD244 | Natural killer cell receptor 2B4 (CD244) |
| CD28 | T-cell-specific surface glycoprotein CD28 (CD28) |
| CD4 | T-cell surface glycoprotein CD4 (CD4) |
| CD40 | CD40L receptor (CD40) |
| CD40-L | CD40 ligand (CD40-L) |
| CD46 | Membrane cofactor protein (CD46) |
| CD5 | T-cell surface glycoprotein CD5 (CD5) |
| CD59 | CD59 glycoprotein (CD59) |
| CD6 | T-cell surface glycoprotein CD6 isoform (CD6) |
| CD83 | CD83 antigen (CD83) |
| CD84 | SLAM family member 5 (CD84) |
| CD93 | Complement component C1q receptor (CD93) |
| CDCP1 | CUB domain-containing protein 1 (CDCP1) |
| CDH1 | Cadherin-1 (CDH1) |
| CDH5 | Cadherin-5 (CDH5) |
| CDSN | Corneodesmosin (CDSN) |
| CEACAM8 | Carcinoembryonic antigenrelated cell adhesion molecule 8 (CEACAM8) |
| CES1 | Liver carboxylesterase 1 (CES1) |
| CFHR5 | Complement factor H-related protein 5 (CFHR5) |
| CHI3L1 | Chitinase-3-like protein 1 (CHI3L1) |
| CHIT1 | Chitotriosidase-1 (CHIT1) |
| CHL1 | Neural cell adhesion molecule L1-like protein (CHL1) |
| CKAP4 | Cytoskeleton-associated protein 4 (CKAP4) |
| CLEC4A | C-type lectin domain family 4 member A (CLEC4A) |
| CLEC4C | C-type lectin domain family 4 member C (CLEC4C) |
| CLEC4D | C-type lectin domain family 4 member D (CLEC4D) |
| CLEC4G | C-type lectin domain family 4 member G (CLEC4G) |
| CLEC6A | C-type lectin domain family 6 member A (CLEC6A) |
| CLEC7A | C-type lectin domain family 7 member A (CLEC7A) |
| CNDP1 | Beta-Ala-His dipeptidase (CNDP1) |
| CNTN1 | Contactin-1 (CNTN1) |
| CNTNAP2 | Contactin-associated protein-like 2 (CNTNAP2) |
| COL18A1 | Collagen alpha-1XVIII chain (COL18A1) |
| COL1A1 | Collagen alpha-1I chain (COL1A1) |
| COMP | Cartilage oligomeric matrix protein (COMP) |
| CPA1 | Carboxypeptidase A1 (CPA1) |
| CPB1 | Carboxypeptidase B (CPB1) |
| CR2 | Complement receptor type 2 (CR2) |
| CRTAC1 | Cartilage acidic protein 1 (CRTAC1) |
| CSF-1 | Macrophage colony-stimulating factor 1 (CSF-1) |
| CST3 | Cystatin-C (CST3) |
| CST5 | Cystatin D (CST5) |
| CSTB | Cystatin-B (CSTB) |
| CTRC | Chymotrypsin C (CTRC) |
| CTSD | Cathepsin D (CTSD) |
| CTSL1 | Cathepsin L1 (CTSL1) |
| CTSZ | Cathepsin Z (CTSZ) |
| CX3CL1 | Fractalkine (CX3CL1) |
| CXADR | Coxsackievirus and adenovirus receptor (CXADR) |
| CXCL1 | C-X-C motif chemokine 1 (CXCL1) |
| CXCL10 | C-X-C motif chemokine 10 (CXCL10) |
| CXCL11 | C-X-C motif chemokine 11 (CXCL11) |
| CXCL12 | Stromal cell-derived factor 1 (CXCL12) |
| CXCL16 | C-X-C motif chemokine 16 (CXCL16) |
| CXCL5 | C-X-C motif chemokine 5 (CXCL5) |
| CXCL6 | C-X-C motif chemokine 6 (CXCL6) |
| CXCL9 | C-X-C motif chemokine 9 (CXCL9) |
| DAPP1 | Dual adapter for phosphotyrosine and 3-phosphotyrosine and 3-phosphoinositide (DAPP1) |
| DCBLD2 | Discoidin, CUB and LCCL domain-containing protein 2 (DCBLD2) |
| DCN | Decorin (DCN) |
| DCTN1 | Dynactin subunit 1 (DCTN1) |
| DDX58 | Probable ATP-dependent RNA helicase DDX58 (DDX58) |
| DECR1 | 2,4-dienoyl-CoA reductase, mitochondrial (DECR1) |
| DEFA1 | Neutrophil defensin 1 (DEFA1) |
| DFFA | DNA fragmentation factor subunit alpha (DFFA) |
| DGKZ | Diacylglycerol kinase zeta (DGKZ) |
| Dkk-1 | Dickkopf-related protein 1 (Dkk-1) |
| DLK-1 | Protein delta homolog 1 (DLK-1) |
| DNER | Delta and Notch-like epidermal growth factor-related receptor (DNER) |
| DPP10 | Inactive dipeptidyl peptidase 10 (DPP10) |
| DPP4 | Dipeptidyl peptidase 4 (DPP4) |
| EDAR | Tumor necrosis factor receptor superfamily member EDAR (EDAR) |
| EFEMP1 | EGF-containing fibulin-like extracellular matrix protein 1 (EFEMP1) |
| EGFR | Epidermal growth factor receptor (EGFR) |
| EGLN1 | Egl nine homolog 1 (EGLN1) |
| EIF4G1 | Eukaryotic translation initiation factor 4 gamma 1 (EIF4G1) |
| EIF5A | Eukaryotic translation initiation factor 5A-1 (EIF5A) |
| ENG | Endoglin (ENG) |
| EN-RAGE | Protein S100-A12 (EN-RAGE) |
| Ep-CAM | Epithelial cell adhesion molecule (Ep-CAM) |
| EPHB4 | Ephrin type-B receptor 4 (EPHB4) |
| F11 | Coagulation factor XI (F11) |
| F7 | Coagulation factor VII (F7) |
| FABP2 | Fatty acid-binding protein, intestinal (FABP2) |
| FABP4 | Fatty acid-binding protein, adipocyte (FABP4) |
| FAM3B | Protein FAM3B (FAM3B) |
| FAP | Prolyl endopeptidase FAP (FAP) |
| FAS | Tumor necrosis factor receptor superfamily member 6 (FAS) |
| FCGR2A | Low affinity immunoglobulin gamma Fc region receptor II-a (FCGR2A) |
| FCGR3B | Low affinity immunoglobulin gamma Fc region receptor III-B (FCGR3B) |
| FCN2 | Ficolin-2 (FCN2) |
| FCRL3 | Fc receptor-like protein 3 (FCRL3) |
| FCRL6 | Fc receptor-like protein 6 (FCRL6) |
| FETUB | Fetuin-B (FETUB) |
| FGF-19 | Fibroblast growth factor 19 (FGF-19) |
| FGF2 | Fibroblast growth factor 2 (FGF2) |
| FGF-21 | Fibroblast growth factor 21 (FGF-21) |
| FGF-23 | Fibroblast growth factor 23 (FGF-23) |
| FGF-5 | Fibroblast growth factor 5 (FGF-5) |
| Flt3L | Fms-related tyrosine kinase 3 ligand (Flt3L) |
| FS | Follistatin (FS) |
| FXYD5 | FXYD domain-containing ion transport regulator 5 (FXYD5) |
| Gal-3 | Galectin-3 (Gal-3) |
| Gal-4 | Galectin-4 (Gal-4) |
| Gal-9 | Galectin-9 (Gal-9) |
| GALNT3 | Polypeptide N-acetylgalactosaminyltransferase 3 (GALNT3) |
| GAS6 | Growth arrest-specific protein 6 (GAS6) |
| GDF-15 | Growth/differentiation factor 15 (GDF-15) |
| GDF-2 | Growth/differentiation factor 2 (GDF-2) |
| GDNF | Glial cell line-derived neurotrophic factor (GDNF) |
| GH | Growth hormone (GH) |
| GIF | Gastric intrinsic factor (GIF) |
| GLB1 | Beta-galactosidase (GLB1) |
| GLO1 | Lactoylglutathione lyase (GLO1) |
| GNLY | Granulysin (GNLY) |
| GP1BA | Platelet glycoprotein Ib alpha chain (GP1BA) |
| GRN | Granulins (GRN) |
| GT | Gastrotropin (GT) |
| HAOX1 | Hydroxyacid oxidase 1 (HAOX1) |
| HB-EGF | Proheparin-binding EGF-like growth factor (HB-EGF) |
| HCLS1 | Hematopoietic lineage cell-specific protein (HCLS1) |
| HEXIM1 | Protein HEXIM1 (HEXIM1) |
| HGF | Hepatocyte growth factor (HGF) |
| HNMT | Histamine N-methyltransferase (HNMT) |
| HO-1 | Heme oxygenase 1 (HO-1) |
| hOSCAR | Osteoclast-associated immunoglobulin-like receptor (hOSCAR) |
| HSD11B1 | Corticosteroid 11-beta-dehydrogenase isozyme 1 (HSD11B1) |
| HSP 27 | Heat shock 27 kDa protein (HSP 27) |
| ICA1 | Islet cell autoantigen 1 (ICA1) |
| ICAM1 | Intercellular adhesion molecule 1 (ICAM1) |
| ICAM-2 | Intercellular adhesion molecule 2 (ICAM-2) |
| ICAM3 | Intercellular adhesion molecule 3 (ICAM3) |
| IDUA | Alpha-L-iduronidase (IDUA) |
| IFN-gamma | Interferon gamma (IFN-gamma) |
| IFNLR1 | Interferon lambda receptor 1 (IFNLR1) |
| IGFBP-1 | Insulin-like growth factor-binding protein 1 (IGFBP-1) |
| IGFBP-2 | Insulin-like growth factor-binding protein 2 (IGFBP-2) |
| IGFBP3 | Insulin-like growth factor-binding protein 3 (IGFBP3) |
| IGFBP6 | Insulin-like growth factor-binding protein 6 (IGFBP6) |
| IGFBP-7 | Insulin-like growth factor-binding protein 7 (IGFBP-7) |
| IgG Fc receptor II-b | Low affinity immunoglobulin gamma Fc region receptor II-b (IgG Fc receptor II-b) |
| IGLC2 | Ig lambda-2 chain C regions (IGLC2) |
| IL-1 alpha | Interleukin-1 alpha (IL-1 alpha) |
| IL10 | Interleukin-10 (IL10) |
| IL-10RA | Interleukin-10 receptor subunit alpha (IL-10RA) |
| IL-10RB | Interleukin-10 receptor subunit beta (IL-10RB) |
| IL-12B | Interleukin-12 subunit beta (IL-12B) |
| IL12RB1 | Interleukin-12 receptor subunit beta-1 (IL12RB1) |
| IL-13 | Interleukin-13 (IL-13) |
| IL-15RA | Interleukin-15 receptor subunit alpha (IL-15RA) |
| IL16 | Pro-interleukin-16 (IL16) |
| IL-17A | Interleukin-17A (IL-17A) |
| IL-17C | Interleukin-17C (IL-17C) |
| IL-17D | Interleukin-17D (IL-17D) |
| IL-17RA | Interleukin-17 receptor A (IL-17RA) |
| IL-18 | Interleukin-18 (IL-18) |
| IL-18BP | Interleukin-18-binding protein (IL-18BP) |
| IL-18R1 | Interleukin-18 receptor 1 (IL-18R1) |
| IL-1ra | Interleukin-1 receptor antagonist protein (IL-1ra) |
| IL1RL2 | Interleukin-1 receptor-like 2 (IL1RL2) |
| IL-1RT1 | Interleukin-1 receptor type 1 (IL-1RT1) |
| IL-1RT2 | Interleukin-1 receptor type 2 (IL-1RT2) |
| IL-2 | Interleukin-2 (IL-2) |
| IL-20 | Interleukin-20 (IL-20) |
| IL-20RA | Interleukin-20 receptor subunit alpha (IL-20RA) |
| IL-22 RA1 | Interleukin-22 receptor subunit alpha-1 (IL-22 RA1) |
| IL-24 | Interleukin-24 (IL-24) |
| IL-27 | Interleukin-27 (IL-27) |
| IL2-RA | Interleukin-2 receptor subunit alpha (IL2-RA) |
| IL-2RB | Interleukin-2 receptor subunit beta (IL-2RB) |
| IL-33 | Interleukin-33 (IL-33) |
| IL-4 | Interleukin-4 (IL-4) |
| IL-4RA | Interleukin-4 receptor subunit alpha (IL-4RA) |
| IL5 | Interleukin-5 (IL5) |
| IL6 | Interleukin-6 (IL6) |
| IL-6RA | Interleukin-6 receptor subunit alpha (IL-6RA) |
| IL-7 | Interleukin-7 (IL-7) |
| IL7R | Interleukin-7 receptor subunit alpha (IL7R) |
| IL-8 | Interleukin-8 (IL-8) |
| IRAK1 | Interleukin-1 receptor-associated kinase 1 (IRAK1) |
| IRAK4 | Interleukin-1 receptor-associated kinase 4 (IRAK4) |
| IRF9 | Interferon regulatory factor 9 (IRF9) |
| ITGA11 | Integrin alpha-11 (ITGA11) |
| ITGA6 | Integrin alpha-6 (ITGA6) |
| ITGAM | Integrin alpha-M (ITGAM) |
| ITGB1BP2 | Melusin (ITGB1BP2) |
| ITGB2 | Integrin beta-2 (ITGB2) |
| ITGB6 | Integrin beta-6 (ITGB6) |
| ITM2A | Integral membrane protein 2A (ITM2A) |
| JAM-A | Junctional adhesion molecule A (JAM-A) |
| JUN | Transcription factor AP-1 (JUN) |
| KIM1 | Kidney Injury Molecule (KIM1) |
| KIT | Mast/stem cell growth factor receptor Kit (KIT) |
| KLK6 | Kallikrein-6 (KLK6) |
| KLRD1 | Natural killer cells antigen CD94 (KLRD1) |
| KPNA1 | Importin subunit alpha-5 (KPNA1) |
| KRT19 | Keratin, type I cytoskeletal 19 (KRT19) |
| LAG3 | Lymphocyte activation gene 3 protein (LAG3) |
| LAMP3 | Lysosome-associated membrane glycoprotein 3 (LAMP3) |
| LAP TGF-beta-1 | Latency-associated peptide transforming growth factor beta-1 (LAP TGF-beta-1) |
| LCN2 | Neutrophil gelatinase-associated lipocalin (LCN2) |
| LDL receptor | Low-density lipoprotein receptor (LDL receptor) |
| LEP | Leptin (LEP) |
| LIF | Leukemia inhibitory factor (LIF) |
| LIF-R | Leukemia inhibitory factor receptor (LIF-R) |
| LILRB1 | Leukocyte immunoglobulin-like receptor subfamily B member 1 (LILRB1) |
| LILRB2 | Leukocyte immunoglobulin-like receptor subfamily B member 2 (LILRB2) |
| LILRB4 | Leukocyte immunoglobulin-like receptor subfamily B member 4 (LILRB4) |
| LILRB5 | Leukocyte immunoglobulin-like receptor subfamily B member 5 (LILRB5) |
| LOX-1 | Lectin-like oxidized LDL receptor 1 (LOX-1) |
| LPL | Lipoproteine lipase (LPL) |
| LTBP2 | Latent-transforming growth factor beta-binding protein 2 (LTBP2) |
| LTBR | Lymphotoxin-beta receptor (LTBR) |
| LY75 | Lymphocyte antigen 75 (LY75) |
| LYVE1 | Lymphatic vessel endothelial hyaluronic acid receptor 1 (LYVE1) |
| MARCO | Macrophage receptor MARCO (MARCO) |
| MASP1 | Mannan-binding lectin serine protease 1 (MASP1) |
| MB | Myoglobin (MB) |
| MBL2 | Mannose-binding protein C (MBL2) |
| MCP-1 | Monocyte chemotactic protein 1 (MCP-1) |
| MCP-2 | Monocyte chemotactic protein 2 (MCP-2) |
| MCP-3 | Monocyte chemotactic protein 3 (MCP-3) |
| MCP-4 | Monocyte chemotactic protein 4 (MCP-4) |
| MEGF9 | Multiple epidermal growth factor-like domains protein 9 (MEGF9) |
| MEPE | Matrix extracellular phosphoglycoprotein (MEPE) |
| MERTK | Tyrosine-protein kinase Mer (MERTK) |
| MET | Hepatocyte growth factor receptor (MET) |
| MFAP5 | Microfibrillar-associated protein 5 (MFAP5) |
| MGMT | Methylated-DNA--protein-cysteine methyltransferase (MGMT) |
| MILR1 | Allergin-1 (MILR1) |
| MMP-1 | Matrix metalloproteinase-1 (MMP-1) |
| MMP-10 | Matrix metalloproteinase-10 (MMP-10) |
| MMP-12 | Matrix metalloproteinase-12 (MMP-12) |
| MMP-2 | Matrix metalloproteinase-2 (MMP-2) |
| MMP-3 | Matrix metalloproteinase-3 (MMP-3) |
| MMP-7 | Matrix metalloproteinase-7 (MMP-7) |
| MMP-9 | Matrix metalloproteinase-9 (MMP-9) |
| MPO | Myeloperoxidase (MPO) |
| NCAM1 | Neural cell adhesion molecule 1 (NCAM1) |
| NCR1 | Natural cytotoxicity triggering receptor 1 (NCR1) |
| NEMO | NF-kappa-B essential modulator (NEMO) |
| NF2 | Merlin (NF2) |
| NFATC3 | Nuclear factor of activated T-cells, cytoplasmic 3 (NFATC3) |
| NID1 | Nidogen-1 (NID1) |
| Notch 3 | Neurogenic locus notch homolog protein 3 (Notch 3) |
| NOTCH1 | Neurogenic locus notch homolog protein 1 (NOTCH1) |
| NRP1 | Neuropilin-1 (NRP1) |
| NRTN | Neurturin (NRTN) |
| NT-3 | Neurotrophin-3 (NT-3) |
| NTF4 | Neurotrophin-4 (NTF4) |
| NT-proBNP | N-terminal prohormone brain natriuretic peptide (NT-proBNP) |
| OPG | Osteoprotegerin (OPG) |
| OPN | Osteopontin (OPN) |
| OSM | Oncostatin-M (OSM) |
| OSMR | Oncostatin-M-specific receptor subunit beta (OSMR) |
| PADI2 | Protein-arginine deiminase type-2 (PADI2) |
| PAI | Plasminogen activator inhibitor 1 (PAI) |
| PAM | Peptidyl-glycine alpha-amidating monooxygenase (PAM) |
| PAPPA | Pappalysin-1 (PAPPA) |
| PAR-1 | Proteinase-activated receptor 1 (PAR-1) |
| PARP-1 | Poly [ADP-ribose] polymerase 1 (PARP-1) |
| PCOLCE | Procollagen C-endopeptidase enhancer 1 (PCOLCE) |
| PCSK9 | Proprotein convertase subtilisin/kexin type 9 (PCSK9) |
| PDGF subunit A | Platelet-derived growth factor subunit A (PDGF subunit A) |
| PDGF subunit B | Platelet-derived growth factor subunit B (PDGF subunit B) |
| PD-L1 | Programmed cell death 1 ligand 1 (PD-L1) |
| PD-L2 | Programmed cell death 1 ligand 2 (PD-L2) |
| PECAM-1 | Platelet endothelial cell adhesion molecule (PECAM-1) |
| PGF | Placenta growth factor (PGF) |
| PGLYRP1 | Peptidoglycan recognition protein 1 (PGLYRP1) |
| PI3 | Elafin (PI3) |
| PIgR | Polymeric immunoglobulin receptor (PIgR) |
| PIK3AP1 | Phosphoinositide 3-kinase adapter protein 1 (PIK3AP1) |
| PLA2G7 | Platelet-activating factor acetylhydrolase (PLA2G7) |
| PLC | Perlecan (PLC) |
| PLTP | Phospholipid transfer protein (PLTP) |
| PLXNA4 | Plexin-A4 (PLXNA4) |
| PLXNB2 | Plexin-B2 (PLXNB2) |
| PON3 | Paraoxonase (PON3) |
| PPP1R9B | Neurabin-2 (PPP1R9B) |
| PRCP | Lysosomal Pro-X carboxypeptidase (PRCP) |
| PRDX1 | Peroxiredoxin-1 (PRDX1) |
| PRDX3 | Thioredoxin-dependent peroxide reductase, mitochondrial (PRDX3) |
| PRDX5 | Peroxiredoxin-5, mitochondrial (PRDX5) |
| PRELP | Prolargin (PRELP) |
| PRKCQ | Protein kinase C theta type (PRKCQ) |
| PROC | Vitamin K-dependent protein C (PROC) |
| Protein BOC | Brother of CDO (Protein BOC) |
| PRSS2 | Trypsin-2 (PRSS2) |
| PRSS27 | Serine protease 27 (PRSS27) |
| PRSS8 | Prostasin (PRSS8 ) |
| PRTN3 | Myeloblastin (PRTN3) |
| PSGL-1 | P-selectin glycoprotein ligand 1 (PSGL-1) |
| PSIP1 | PC4 and SFRS1-interacting protein (PSIP1) |
| PSP-D | Pulmonary surfactant-associated protein D (PSP-D) |
| PTH1R | Parathyroid hormone/parathyroid hormone-related peptide receptor (PTH1R) |
| PTPRS | Receptor-type tyrosine-protein phosphatase S (PTPRS) |
| PTX3 | Pentraxin-related protein PTX3 (PTX3) |
| QPCT | Glutaminyl-peptide cyclotransferase (QPCT) |
| RAGE | Receptor for advanced glycosylation end products (RAGE) |
| RARRES2 | Retinoic acid receptor responder protein 2 (RARRES2) |
| REG1A | Lithostathine-1-alpha (REG1A) |
| REG3A | Regenerating islet-derived protein 3-alpha (REG3A) |
| REN | Renin (REN) |
| RETN | Resistin (RETN) |
| SAA4 | Serum amyloid A-4 protein (SAA4) |
| SCF | Stem cell factor (SCF) |
| SCGB3A2 | Secretoglobin family 3A member 2 (SCGB3A2) |
| SELE | E-selectin (SELE) |
| SELL | L-selectin (SELL) |
| SELP | P-selectin (SELP) |
| SERPINA12 | Serpin A12 (SERPINA12) |
| SERPINA5 | Plasma serine protease inhibitor (SERPINA5) |
| SERPINA7 | Thyroxine-binding globulin (SERPINA7) |
| SH2B3 | SH2B adapter protein 3 (SH2B3) |
| SH2D1A | SH2 domain-containing protein 1A (SH2D1A) |
| SHPS-1 | Tyrosine-protein phosphatase non-receptor type substrate 1 (SHPS-1) |
| SIRT2 | SIR2-like protein 2 (SIRT2) |
| SIT1 | Signaling threshold-regulating transmembrane adapter 1 (SIT1) |
| SLAMF1 | Signaling lymphocytic activation molecule (SLAMF1) |
| SLAMF7 | SLAM family member 7 (SLAMF7) |
| SOD1 | Superoxide dismutase [Cu-Zn] (SOD1) |
| SOD2 | Superoxide dismutase [Mn], mitochondrial (SOD2) |
| SORT1 | Sortilin (SORT1) |
| SPARCL1 | SPARC-like protein 1 (SPARCL1) |
| SPON1 | Spondin-1 (SPON1) |
| SPON2 | Spondin-2 (SPON2) |
| SPRY2 | Protein sprouty homolog 2 (SPRY2) |
| SRC | Proto-oncogene tyrosine-protein kinase Src (SRC) |
| SRPK2 | SRSF protein kinase 2 (SRPK2) |
| ST1A1 | Sulfotransferase 1A1 (ST1A1) |
| ST2 | ST2 protein (ST2) |
| ST6GAL1 | Beta-galactoside alpha-2,6-sialyltransferase 1 (ST6GAL1) |
| STAMBP | STAM-binding protein (STAMBP) |
| STC1 | Stanniocalcin-1 (STC1) |
| STK4 | Serine/threonine-protein kinase 4 (STK4) |
| TANK | TRAF family member-associated NF-kappa-B activator (TANK) |
| TCN2 | Transcobalamin-2 (TCN2) |
| TF | Tissue factor (TF) |
| TFF3 | Trefoil factor 3 (TFF3) |
| TFPI | Tissue factor pathway inhibitor (TFPI) |
| TGF-alpha | Transforming growth factor alpha (TGF-alpha) |
| TGFBI | Transforming growth factor-beta-induced protein ig-h3 (TGFBI) |
| TGFBR3 | Transforming growth factor beta receptor type 3 (TGFBR3) |
| TGM2 | Protein-glutamine gamma-glutamyltransferase 2 (TGM2) |
| THBS2 | Thrombospondin-2 (THBS2) |
| THBS4 | Thrombospondin-4 (THBS4) |
| THPO | Thrombopoietin (THPO) |
| TIE1 | Tyrosine-protein kinase receptor Tie-1 (TIE1) |
| TIE2 | Angiopoietin-1 receptor (TIE2) |
| TIMD4 | T-cell immunoglobulin and mucin domain-containing protein 4 (TIMD4) |
| TIMP1 | Metalloproteinase inhibitor 1 (TIMP1) |
| TIMP4 | Metalloproteinase inhibitor 4 (TIMP4) |
| TLT-2 | Trem-like transcript 2 protein (TLT-2) |
| TM | Thrombomodulin (TM) |
| TNC | Tenascin (TNC) |
| TNF | Tumor necrosis factor alpha (TNF) |
| TNFB | TNF-beta (TNFB) |
| TNF-R1 | Tumor necrosis factor receptor 1 (TNF-R1) |
| TNF-R2 | Tumor necrosis factor receptor 2 (TNF-R2) |
| TNFRSF10A | Tumor necrosis factor receptor superfamily member 10A (TNFRSF10A) |
| TNFRSF10C | Tumor necrosis factor receptor superfamily member 10C (TNFRSF10C) |
| TNFRSF11A | Tumor necrosis factor receptor superfamily member 11A (TNFRSF11A) |
| TNFRSF13B | Tumor necrosis factor receptor superfamily member 13B (TNFRSF13B) |
| TNFRSF14 | Tumor necrosis factor receptor superfamily member 14 (TNFRSF14) |
| TNFRSF9 | Tumor necrosis factor receptor superfamily member 9 (TNFRSF9) |
| TNFSF13B | Tumor necrosis factor ligand superfamily member 13B (TNFSF13B) |
| TNFSF14 | Tumor necrosis factor ligand superfamily member 14 (TNFSF14) |
| TNXB | Tenascin-X (TNXB) |
| t-PA | Tissue-type plasminogen activator (t-PA) |
| TPSAB1 | Tryptase alpha/beta-1 (TPSAB1) |
| TR | Transferrin receptor protein 1 (TR) |
| TRAF2 | TNF receptor-associated factor 2 (TRAF2) |
| TRAIL | TNF-related apoptosis-inducing ligand (TRAIL) |
| TRAIL-R2 | TNF-related apoptosis-inducing ligand receptor 2 (TRAIL-R2) |
| TRANCE | TNF-related activation-induced cytokine (TRANCE) |
| TR-AP | Tartrate-resistant acid phosphatase type 5 (TR-AP) |
| TREM1 | Triggering receptor expressed on myeloid cells 1 (TREM1) |
| TRIM21 | E3 ubiquitin-protein ligase TRIM21 (TRIM21) |
| TRIM5 | Tripartite motif-containing protein 5 (TRIM5) |
| TSLP | Thymic stromal lymphopoietin (TSLP) |
| TWEAK | Tumor necrosis factor Ligand superfamily, member 12 (TWEAK) |
| UMOD | Uromodulin (UMOD) |
| uPA | Urokinase-type plasminogen activator (uPA) |
| U-PAR | Urokinase plasminogen activator surface receptor (U-PAR) |
| VASN | Vasorin (VASN) |
| VCAM1 | Vascular cell adhesion protein 1 (VCAM1) |
| VEGF-A | Vascular endothelial growth factor A (VEGF-A) |
| VEGFD | Vascular endothelial growth factor D (VEGFD) |
| VSIG2 | V-set and immunoglobulin domain-containing protein 2 (VSIG2) |
| vWF | von Willebrand factor (vWF) |
| XCL1 | Lymphotactin (XCL1) |
| ZBTB16 | Zinc finger and BTB domain-containing protein 16 (ZBTB16) |

**Table S2.** Model estimates for LASSO-regularized logistic regression for the acute VTE event model.

| Model information |  |
| --- | --- |
| R² | 0.16 |
| 10-fold cross-validated R² | 0.02 |
| Optimal λ | 1.88 |
| Non-linear term penalty | 1.025 |
| Sample size | 549 |
| Number of covariates | 503 |
| Number of selected variables | 23 |
| Number of protein selected | 21 |

| **Variable Name** | **Gene Name** | **Transformation** | | **Direction** | **Lambda Ratio** |
| --- | --- | --- | --- | --- | --- |
| P03951 | F11 | ^3 | | + | 2.36796003812897 |
| Q9HCB6 | SPON1 | | ^2 | - | 1.5211855087002 |
| O15169 | AXIN1 | | log | - | 1.45936579112829 |
| P10747 | CD28 | | ^3 | - | 1.40005837561212 |
| O14867 | BACH1 | | ^-0.5 | + | 1.39361926749318 |
| Q6ZUJ8 | PIK3AP1 | | ^-2 | + | 1.38082975873416 |
| P01374 | LTA | | ^-2 | + | 1.37447908631562 |
| P31994 | FCGR2B | | log | - | 1.35560177932764 |
| Q06141 | REG3A | | log | - | 1.33083471911909 |
| P30048 | PRDX3 | | ^0.5 | - | 1.31862139608747 |
| P01833 | PIGR | | ^-2 | + | 1.30652015704143 |
| P14778 | IL1R1 | | ^3 | - | 1.28857620843136 |
| Q9NWZ3 | IRAK4 | | log | - | 1.27087870476895 |
| Q06830 | PRDX1 | | ^-2 | + | 1.24765954861354 |
| FXa Inhibitors | - | | ^1 | - | 1.24192134878624 |
| Q14435 | GALNT3 | | ^-0.5 | - | 1.22486461013566 |
| Q14767 | LTBP2 | | log | + | 1.16968270130262 |
| P35475 | IDUA | | ^-2 | + | 1.11184960310384 |
| P16871 | IL7R | | ^-2 | - | 1.10164594808176 |
| P56470 | LGALS4 | | ^-2 | + | 1.05687597033405 |
| Platelet count | - | | log | + | 1.02804473167953 |
| Q99075 | HBEGF | | ^-0.5 | - | 1 |
| Q9HB29 | IL1RL2 | | ^-0.5 | - | 1 |

**Table S3.** Model estimates for LASSO-regularized logistic regression for the 12 months follow up model.

| Model information |  |
| --- | --- |
| R² | 0.81 |
| 10-fold cross-validated R² | 0.11 |
| Optimal λ | 0.951 |
| Non-linear term penalty | 1.025 |
| Sample size | 184 |
| Number of covariates | 503 |
| Number of selected variables | 74 |
| Number of proteins selected | 66 |

| **Variable name** | **Gene name** | **Transformation** | **Direction** | **Lambda ratio** |
| --- | --- | --- | --- | --- |
| Q06830 | PRDX1 | log | - | 6.61943327671832 |
| C09 | - | ^1 | - | 4.92824945565546 |
| Q13332 | PTPRS | ^2 | - | 4.83820955392895 |
| P03951 | F11 | ^3 | + | 4.45295841316076 |
| Q8IYS5 | OSCAR | ^3 | - | 4.43247849520095 |
| Q7Z6M3 | MILR1 | ^-2 | + | 4.23278897670022 |
| P01130 | LDLR | ^3 | + | 3.73742567068778 |
| Q14116 | IL18 | ^-2 | + | 3.73742567068778 |
| Platelet count | - | ^2 | + | 3.09378752088411 |
| P35225 | IL13 | ^2 | + | 2.94082012442504 |
| P35218 | CA5A | ^3 | + | 2.90043044891966 |
| P09603 | CSF1 | ^3 | + | 2.88709087281528 |
| Q9P0M4 | IL17C | ^3 | + | 2.8343432631817 |
| P01833 | PIGR | ^3 | - | 2.75702328492184 |
| P39905 | GDNF | ^-2 | - | 2.7191579034543 |
| P01375 | TNF | ^-2 | + | 2.65720106753591 |
| O15467 | CCL16 | ^-2 | - | 2.52581999290637 |
| Q96F46 | IL17RA | ^-0.5 | + | 2.49112999298043 |
| Q9HD89 | RETN | ^3 | - | 2.4120281767139 |
| Q12933 | TRAF2 | ^3 | - | 2.40093484625974 |
| Q8N423 | LILRB2 | ^2 | - | 2.40093484625974 |
| Inflammatory disease | - | ^1 | - | 2.32469703144802 |
| P01583 | IL1A | ^3 | + | 2.30336284517542 |
| P15086 | CPB1 | ^-2 | + | 2.27172810590146 |
| BMI (kg/m^2^) | - | ^-0.5 | - | 2.26128003948828 |
| P39900 | MMP12 | ^-2 | - | 2.16938349393703 |
| Q9UHF4 | IL20RA | ^-0.5 | - | 2.13958884766162 |
| Erythrocyte count | - | ^-2 | + | 2.1297485123155 |
| P08254 | MMP3 | ^3 | - | 2.11995343435602 |
| Q14767 | LTBP2 | ^-2 | + | 2.0526377312291 |
| Q6EMK4 | VASN | ^-2 | + | 1.98745952971614 |
| P10721 | KIT | ^-2 | - | 1.9783188631434 |
| Q13231 | CHIT1 | ^-2 | - | 1.95114832786825 |
| P04275 | VWF | ^-2 | - | 1.94217466272823 |
| Q9BYF1 | ACE2 | ^-2 | - | 1.89792162514235 |
| Q9BQ51 | PDCD1LG2 | ^3 | - | 1.86324629505809 |
| Q969D9 | TSLP | ^3 | + | 1.84614692985361 |
| Q8IW75 | SERPINA12 | ^-2 | - | 1.76297536074093 |
| P16278 | GLB1 | log | - | 1.69914415892686 |
| Q9Y5C1 | ANGPTL3 | ^2 | + | 1.6835507901996 |
| Q14393 | GAS6 | ^3 | - | 1.66042864493535 |
| Q14435 | GALNT3 | ^-0.5 | + | 1.60031030278893 |
| P55773 | CCL23 | ^-2 | - | 1.58562395113459 |
| Q8N608 | DPP10 | ^-1 | + | 1.58562395113459 |
| P17936 | IGFBP3 | ^3 | + | 1.57833139517037 |
| Q14790 | CASP8 | ^-2 | - | 1.53527501920254 |
| O14788 | TNFSF11 | ^3 | - | 1.51418931616273 |
| P60568 | IL2 | ^0.5 | - | 1.50029331316278 |
| P59665 | DEFA1 | ^-1 | - | 1.48652483642213 |
| P63241 | EIF5A | ^3 | + | 1.45265391805368 |
| P02786 | TFRC | log | + | 1.38720977393467 |
| O14786 | NRP1 | ^3 | - | 1.38082975873416 |
| P11215 | ITGAM | ^-2 | + | 1.3681576217266 |
| O43736 | ITM2A | ^-2 | + | 1.35560177932764 |
| Q8NEV9 | IL27 | ^3 | + | 1.33083471911909 |
| Q9NP99 | TREM1 | ^2 | - | 1.30652015704143 |
| Arterial hypertension | - | ^1 | - | 1.30051124720074 |
| P26022 | PTX3 | ^0.5 | + | 1.28264982588181 |
| Q06141 | REG3A | ^-2 | + | 1.27087870476895 |
| Immobilization | - | ^1 | - | 1.23620953992216 |
| O95750 | FGF19 | ^-2 | + | 1.19695569922761 |
| Q13490 | BIRC2 | ^3 | - | 1.18051652571795 |
| P13501 | CCL5 | ^3 | - | 1.15894830095202 |
| Q9UHC6 | CNTNAP2 | ^1 | - | 1.15894830095202 |
| P05112 | IL4 | ^3 | - | 1.11184960310384 |
| D-dimer (ng/mL) | - | ^1 | - | 1.10164594808176 |
| O75015 | FCGR3B | ^3 | - | 1.08651577334061 |
| P16871 | IL7R | ^-2 | - | 1.08151870131895 |
| Q96D42 | HAVCR1 | ^3 | + | 1.07654461168686 |
| P80188 | LCN2 | ^3 | + | 1.05687597033405 |
| Q9UBR2 | CTSZ | ^-2 | + | 1.0423606733471 |
| P09038 | FGF2 | ^3 | - | 1.02804473167953 |
| P12034 | FGF5 | ^-2 | - | 1.01861016988266 |
| P41159 | LEP | ^-2 | - | 1.01861016988266 |

**Table S4.** Model estimates for LASSO-regularized logistic regression for the baseline model, excluding individual under DOACs treatment.

| Model information |  |
| --- | --- |
| R² | 0.30 |
| 10-fold cross-validated R² | 0.01 |
| Optimal λ | 2.895 |
| Non-linear term penalty | 1.025 |
| Sample size | 397 |
| Number of covariates | 501 |
| Number of selected variables | 49 |
| Number of proteins selected | 26 |

| **Variable name** | **Gene name** | **Transformation** | **Direction** | **Lambda ratio** |
| --- | --- | --- | --- | --- |
| Q9HCB6 | SPON1 | ^3 | - | 2.01513571326856 |
| P03951 | F11 | ^3 | + | 1.96016345512164 |
| Q07325 | CXCL9 | log | - | 1.86324629505809 |
| Q9UKX5 | ITGA11 | log | + | 1.83765618411626 |
| Coronary artery disease | - | ^1 | - | 1.69132950409563 |
| C09 | - | ^1 | - | 1.54236863657103 |
| P01374 | LTA | ^-2 | + | 1.5211855087002 |
| Q14435 | GALNT3 | log | + | 1.51418931616273 |
| Q9NWZ3 | IRAK4 | log | - | 1.50722530031229 |
| O75475 | PSIP1 | ^0.5 | - | 1.47968805424937 |
| Q06830 | PRDX1 | ^-2 | + | 1.43932263709185 |
| P20783 | NTF3 | ^3 | - | 1.40005837561212 |
| O14798 | TNFRSF10C | ^-1 | - | 1.38720977393467 |
| Q8TAD2 | IL17D | log | + | 1.38720977393467 |
| P63241 | EIF5A | ^2 | - | 1.30652015704143 |
| P10747 | CD28 | ^3 | - | 1.28857620843136 |
| P22748 | CA4 | ^-0.5 | - | 1.25921560947067 |
| Q06141 | REG3A | ^-2 | + | 1.25342426134142 |
| Platelet count | - | log | + | 1.20804213135482 |
| P18564 | ITGB6 | ^3 | + | 1.20248613881085 |
| Gender (Female) | - | ^1 | + | 1.18597100939409 |
| P31994 | FCGR2B | log | - | 1.16968270130262 |
| P35475 | IDUA | ^-2 | + | 1.09657928979661 |
| Q9NQ25 | SLAMF7 | ^-2 | - | 1.09657928979661 |
| Hx. of DVT | - | ^1 | + | 1.08151870131895 |
| Q6ZUJ8 | PIK3AP1 | ^-1 | + | 1.0666649572778 |
| Q99075 | HBEGF | ^-2 | - | 1.04717681878306 |
| P19474 | TRIM21 | ^1 | - | 1.03279473143398 |
| P07451 | CA3 | ^3 | - | 1.01392540735477 |
| P01375 | TNF | ^3 | + | 1.00926219085164 |
| Q03431 | PTH1R | ^-0.5 | + | 1.00462042127942 |

**Table S5.** Model estimates for LASSO-regularized logistic regression for the 12 months follow up model, excluding individual under DOACs treatment.

| Model information |  |
| --- | --- |
| R² | 0.64 |
| 10-fold cross-validated R² | 0.25 |
| Optimal λ | 0.92 |
| Non-linear term penalty | 1.025 |
| Sample size | 76 |
| Number of covariates | 501 |
| Number of selected variables | 17 |
| Number of proteins selected | 16 |

| **Variable name** | **Gene name** | **Transformation** | **Direction** | **Lambda ratio** |
| --- | --- | --- | --- | --- |
| Q13332 | PTPRS | ^1 | - | 3.23974257411015 |
| P03951 | F11 | ^-2 | - | 2.21996609335716 |
| Q9NPY3 | CD93 | ^-2 | + | 2.03380028483681 |
| Q13007 | IL24 | ^3 | + | 1.85467690641324 |
| Q14116 | IL18 | ^-2 | + | 1.65279204937993 |
| Q15166 | PON3 | ^3 | - | 1.53527501920254 |
| P80370 | DLK1 | ^0.5 | - | 1.38720977393467 |
| Q9BXR6 | CFHR5 | ^3 | + | 1.38082975873416 |
| P08254 | MMP3 | ^-2 | + | 1.34316116427957 |
| BMI (kg/m^2^) | - | ^-1 | - | 1.22486461013566 |
| Q96DB9 | FXYD5 | ^-2 | + | 1.18597100939409 |
| Q12884 | FAP | ^2 | - | 1.17508712814589 |
| P16278 | GLB1 | ^3 | - | 1.16968270130262 |
| Q6EMK4 | VASN | ^3 | - | 1.09657928979661 |
| Q06141 | REG3A | ^-2 | + | 1.09153593394018 |
| P35218 | CA5A | ^3 | + | 1.02804473167953 |
| P00813 | ADA | ^-2 | + | 1 |

**Table S6.** Model estimates for LASSO-regularized logistic regression for the baseline model, excluding individual with elongated APTT and normal or high FXI:C.

| Model information |  |
| --- | --- |
| R² | 0.23 |
| 10-fold cross-validated R² | 0.014 |
| Optimal λ | 3.74 |
| Non-linear term penalty | 1.025 |
| Sample size | 476 |
| Number of covariates | 503 |
| Number of selected variables | 27 |
| Number of proteins selected | 22 |

| **Variable name** | **Gene name** | **Transformation** | **Direction** | **Lambda ratio** |
| --- | --- | --- | --- | --- |
| P03951 | F11 | ^3 | + | 2.56099306521241 |
| P14778 | IL1R1 | ^3 | - | 1.66042864493535 |
| P01833 | PIGR | ^-2 | + | 1.63762406276734 |
| FXa Inhibitors | - | ^1 | - | 1.5211855087002 |
| P10747 | CD28 | ^3 | - | 1.50722530031229 |
| Q9NWZ3 | IRAK4 | log | - | 1.50029331316278 |
| C-reactive protein (mg/mL) | - | log | + | 1.44597291403222 |
| P01374 | LTA | ^-2 | + | 1.43270294591346 |
| Q6ZUJ8 | PIK3AP1 | ^-2 | + | 1.40652723512323 |
| Chronic liver disease | - | ^1 | - | 1.38082975873416 |
| Q06830 | PRDX1 | ^-2 | + | 1.3681576217266 |
| O95786 | DDX58 | ^-2 | - | 1.34316116427957 |
| Q14435 | GALNT3 | ^-2 | - | 1.34316116427957 |
| Q06141 | REG3A | log | - | 1.3369837361747 |
| Q9HCB6 | SPON1 | ^3 | - | 1.3369837361747 |
| O14867 | BACH1 | ^-0.5 | + | 1.32471398244545 |
| Q14767 | LTBP2 | log | + | 1.28264982588181 |
| P00749 | PLAU | ^-2 | - | 1.20248613881085 |
| P16871 | IL7R | ^-2 | - | 1.19695569922761 |
| Platelet count | - | ^-0.5 | - | 1.16430313034348 |
| P31994 | FCGR2B | log | - | 1.15361809933751 |
| Q9HB29 | IL1RL2 | ^-0.5 | - | 1.1273325617443 |
| Q9NQ25 | SLAMF7 | ^-2 | - | 1.1273325617443 |
| P42785 | PRCP | ^1 | + | 1.12214776632611 |
| P35475 | IDUA | ^-2 | + | 1.05201521683988 |
| P18564 | ITGB6 | ^3 | + | 1.02804473167954 |
| Female sex | - | ^1 | + | 1.00462042127942 |

**Table S7.** Model estimates for LASSO-regularized logistic regression for 12 months follow up model, excluding individual with elongated aPTT and normal or high FXI:C.

| Model information |  |
| --- | --- |
| R² | 0.652 |
| 10-fold cross-validated R² | 0.064 |
| Optimal λ | 3.756 |
| Non-linear term penalty | 1.025 |
| Sample size | 164 |
| Number of covariates | 503 |
| Number of selected variables | 29 |
| Number of proteins selected | 24 |

| **Variable name** | **Gene name** | **Transformation** | **Direction** | **Lambda ratio** |
| --- | --- | --- | --- | --- |
| Q06830 | PRDX1 | log | - | 3.28485730917922 |
| C09 | - | ^1 | - | 2.58471347175214 |
| P03951 | F11 | ^3 | + | 2.57282593206736 |
| Q7Z6M3 | MILR1 | ^-2 | + | 2.26128003948828 |
| P01130 | LDLR | ^3 | + | 2.10049821896839 |
| Q8IYS5 | OSCAR | ^3 | - | 2.05263773122909 |
| Q14116 | IL18 | ^-2 | + | 1.98745952971614 |
| Q14435 | GALNT3 | ^-2 | + | 1.91550053745587 |
| Q9UKX5 | ITGA11 | ^-2 | + | 1.89792162514235 |
| Q13332 | PTPRS | ^1 | - | 1.86324629505808 |
| Q9NYY1 | IL20 | ^3 | + | 1.7148819564113 |
| Q96F46 | IL17RA | ^1 | - | 1.69914415892686 |
| P39905 | GDNF | ^-2 | - | 1.66042864493535 |
| Platelet count | - | ^1 | + | 1.57833139517036 |
| O15467 | CCL16 | ^-2 | - | 1.46610867588408 |
| P01375 | TNF | ^-2 | + | 1.43932263709185 |
| Erythrocyte count | - | ^-2 | + | 1.34316116427956 |
| Q14767 | LTBP2 | ^-2 | + | 1.25342426134142 |
| O14786 | NRP1 | ^2 | - | 1.24765954861354 |
| Q9UQV4 | LAMP3 | ^-2 | + | 1.22486461013566 |
| Q6EMK4 | VASN | ^-2 | + | 1.19145069508267 |
| P35218 | CA5A | ^3 | + | 1.15894830095202 |
| P02786 | TFRC | ^-1 | - | 1.11184960310384 |
| P22749 | GNLY | ^2 | + | 1.11184960310384 |
| BMI (kg/m^2^) | - | log | + | 1.10673601646267 |
| Inflammatory disease | - | ^1 | - | 1.10164594808176 |
| P39900 | MMP12 | ^-2 | - | 1.06175918255709 |
| P04275 | VWF | ^-2 | - | 1.03756667818838 |
| P35225 | IL13 | ^2 | + | 1.02331657798702 |

**Table S8.** Proteins with high correlations (Spearman’s rank correlation coefficient>0.8) with proteins selected in the baseline model.

| Selected protein | Correlated proteins (correlation coefficient) |
| --- | --- |
| AXIN1 | STAMBP: 0.920, SIRT2: 0.894, EIF4G1: 0.839, SH2B3:0.832, PPP1R9B: 0.831, STK4: 0.824, IKBKG: 0.822, CASP3: 0.813, ITGB1BP2: 0.806, DAPP1: 0.803 |
| HBEGF | PDGFB: 0.802 |
| IRAK4 | DAPP1: 0.923, EIF4G1: 0.919, SH2B3: 0.908, IRAK1: 0.902, ICA1: 0.897, HCLS1: 0.887, SRPK2: 0.884, PPP1R9B: 0.883, PLXNA4: 0.878, HEXIM1: 0.870, DCTN1: 0.856, SPRY2: 0.844, ITGB1BP2: 0.837, CASP3: 0.827, IKBKG: 0.826, SIRT2p: 0.822, STK4: 0.810, STAMBP: 0.808, NF2: 0.807 |
| BACH1 | IRAK1: 0.891, SRPK2: 0.887, EIF4G1: 0.886, HEXIM1: 0.875, HCLS1: 0.865, SH2B3: 0.865, PPP1R9B: 0.859, ICA1: 0.851, DAPP1: 0.836, SPRY2: 0.818, PLXNA4: 0.813, TANK: 0.803, TRIM5: 0.803, DCTN1: 0.802, IKBKG: 0.801 |

**Table ST9.** Proteins with high correlations (Spearman’s rank correlation coefficient>0.8) with proteins selected in the 12 months follow up model.

| Selected protein | Correlated proteins (correlation coefficient) |
| --- | --- |
| CPB1 | CPA1: 0.881, PRSS2: 0.855 |
| HBEGF | PDGFB: 0.802 |
| CCL5 | CXCL1: 0.867, CXCL5: 0.864 |
| RETN | PGLYRP1: 0.865 |
| TRAF2 | DCTN1: 0.888, SIRT2: 0.884, EIF4G1: 0.877, HEXIM1: 0.876, STAMBP: 0.856, IRAK1: 0.855 |

**Figure S1.** Factor XI activity (FXI:C) and expression of the associated protein signature in patients with and without chronic liver disease.

**
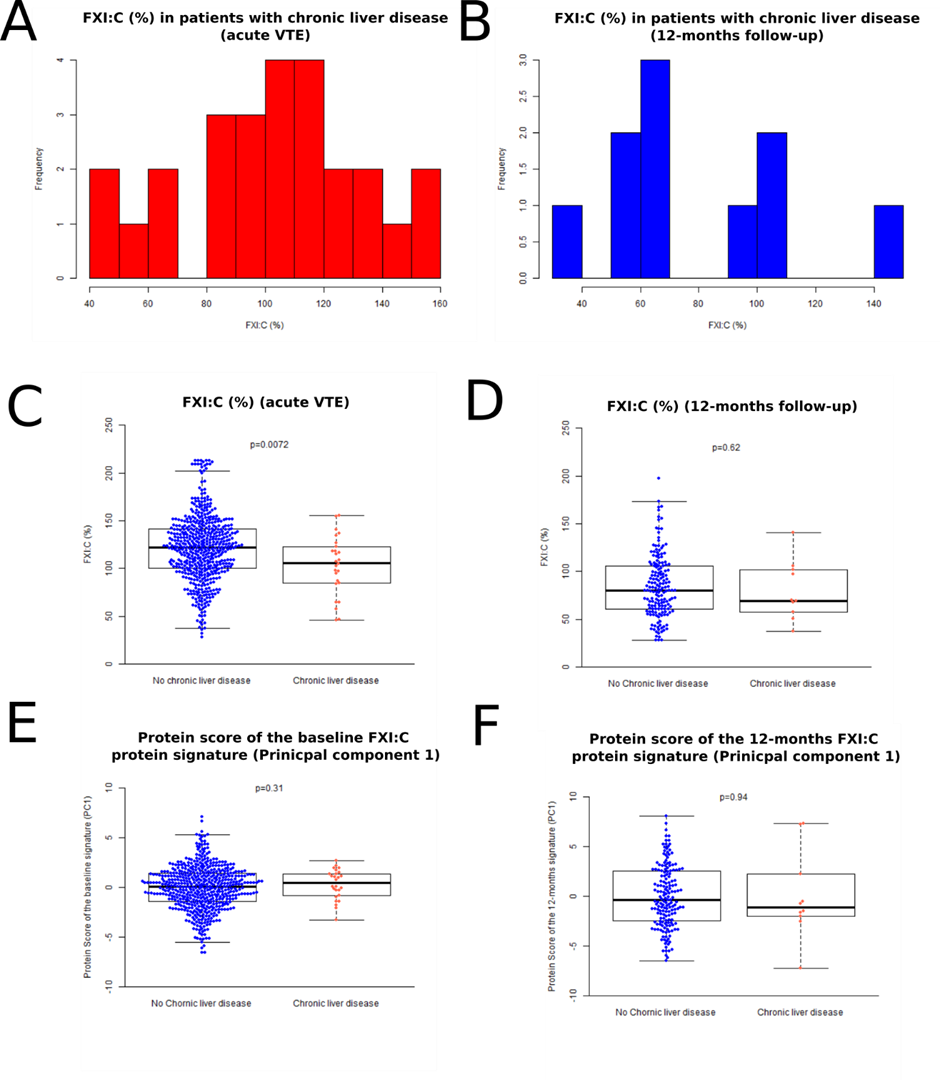
**

**Figure S2.** Protein-protein interaction network of the proteins selected by LASSO regression in the baseline model.
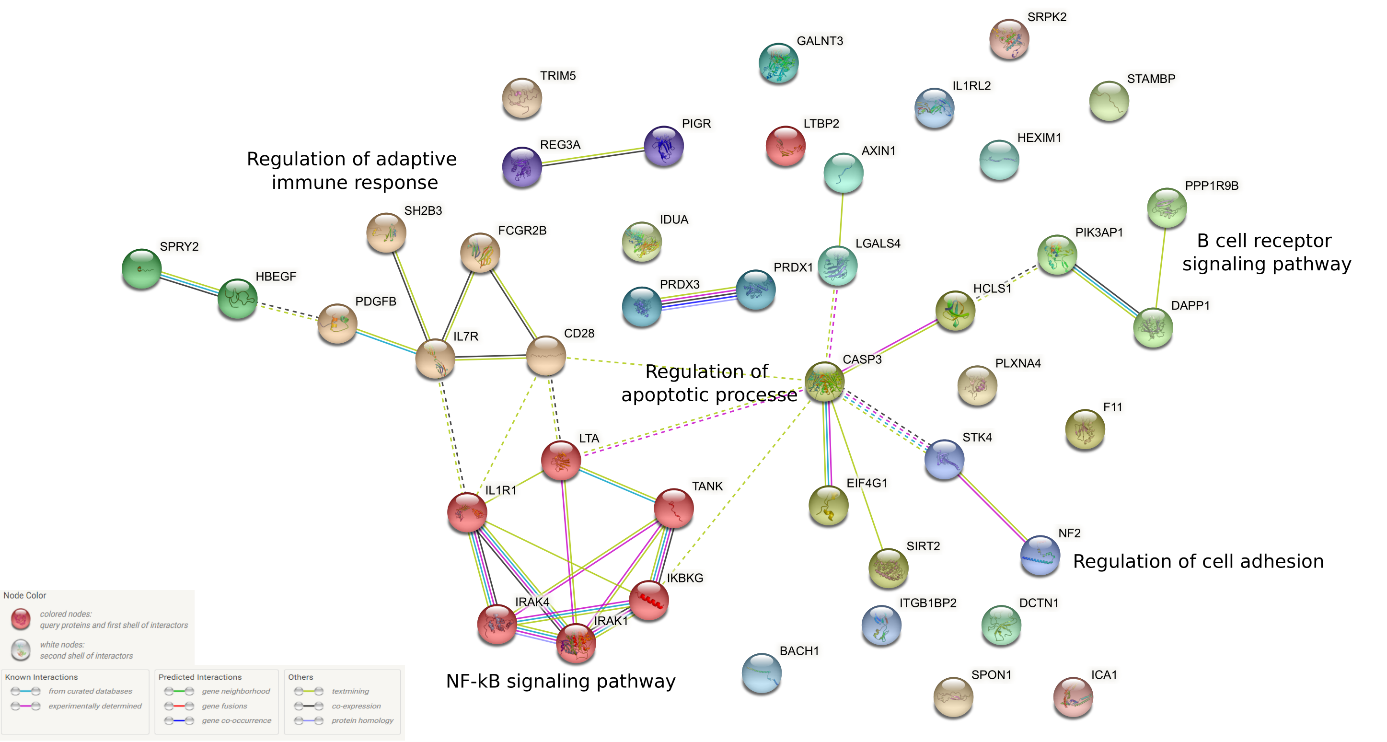


STRING network analysis incorporating proteins selected by LASSO regression in the baseline, and highly correlated (Spearman’s p>0.8) proteins with the selected proteins in the model. Non-connected proteins were excluded from the representation. Node colors represent the cluster membership. These cluster were assessed using Markov clustering algorithm with the default value inflation parameter of three. Dashed lines show inter cluster connection and solid lines represent intra cluster connections. Protein-protein connections (i.e., the edges of the graph) reflect experimentally established protein-protein interactions, co-expression patterns, protein homology, and putative connections based on text mining. Figure created with STRING database (version 11.5, https://string-db.org/).

**Figure S3.** Protein-protein interaction network of the proteins selected by LASSO regression in the 12 months follow up model.


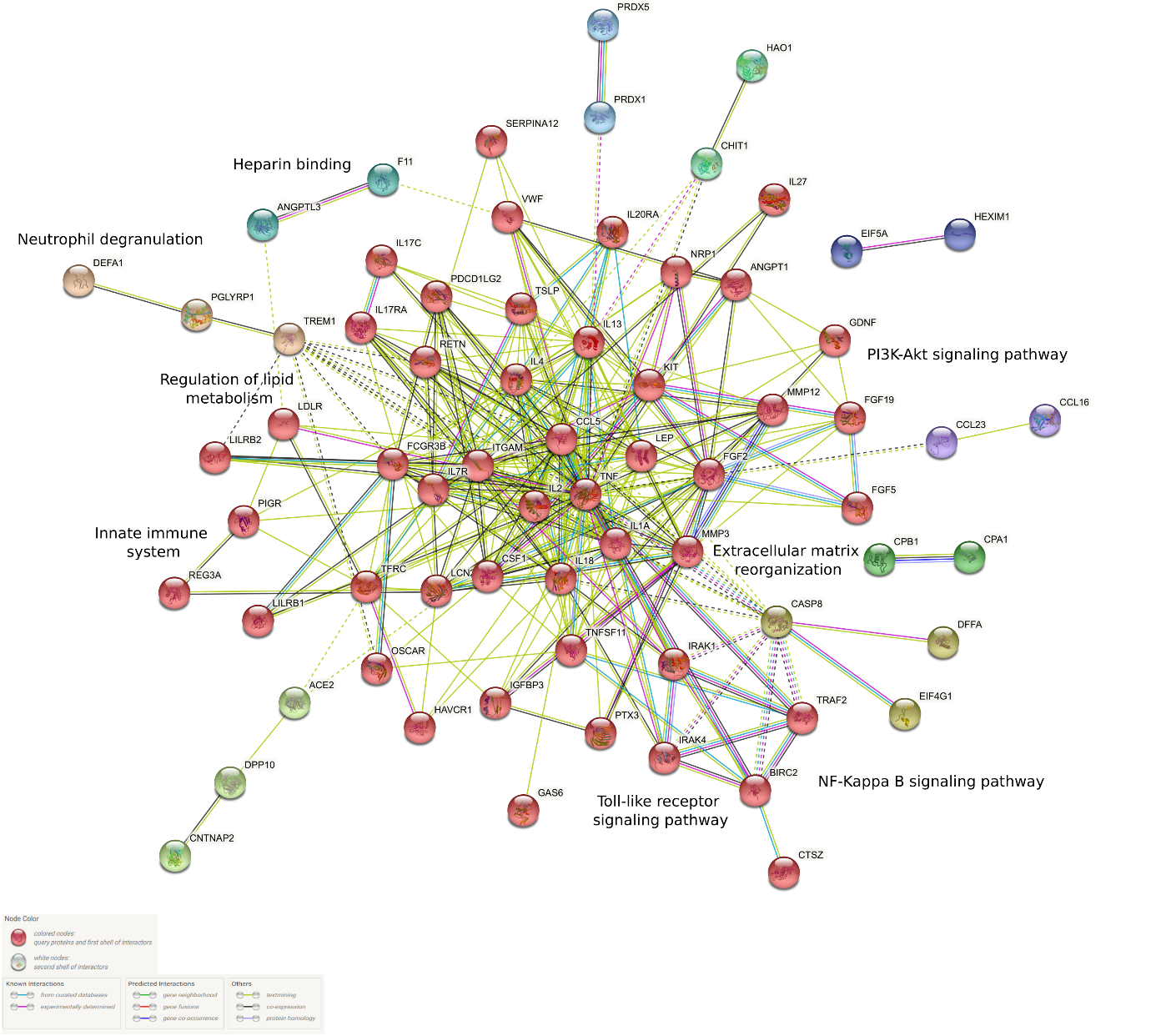


STRING network analysis incorporating proteins selected by LASSO regression in the 12 months follow up model, and highly correlated (Spearman’s p>0.8) proteins with the selected proteins in the model. Non-connected proteins were excluded from the representation. Node colors represent the cluster membership. These cluster were assessed using Markov clustering algorithm with the default value inflation parameter of three. Dashed lines show inter cluster connection and solid lines represent intra cluster connections. Protein-protein connections (i.e., the edges of the graph) reflect experimentally established protein-protein interactions, co-expression patterns, protein homology, and putative connections based on text mining. Figure created with STRING database (version 11.5, https://string-db.org/).
